# Biology of host-dependent restriction-modification in prokaryotes

**DOI:** 10.1128/ecosalplus.esp-0014-2022

**Published:** 2025-08-26

**Authors:** Brian P. Anton, Robert Blumenthal, James B. Eaglesham, Iwona Mruk, Richard J. Roberts, Shuang-yong Xu, Peter R. Weigele, Elisabeth A. Raleigh

**Affiliations:** 1 New England Biolabs Inc. 1696 , Ipswich, Massachusetts, USA; 2 University of Toledo 7923, Toledo, Ohio, USA; 3 Department of Microbiology, Faculty of Biology, University of Gdansk, Gdansk, Poland; National Institutes of Health, Bethesda, Maryland, USA

**Keywords:** host-dependent restriction-modification, restriction endonuclease, anti-phage, genome defense, mobile DNA, bacteriophages, DNA methylation, defense islands, DNA phosporothioation

## Abstract

Understanding the mechanisms that modulate horizontal genetic exchange in prokaryotes is a key problem in biology. DNA entry is limited by resident host-dependent restriction-modification (RM) systems (HDRM), which are present in most prokaryotic genomes. This review specifically focuses on the biological functions of HDRM, rather than detailed enzyme mechanisms. DNA in each cell carries epigenetic marks imposed by host-modifying enzymes (HDM), most often not only base methylation but also additions to the phosphodiester backbone. The pattern of base and backbone modifications is read by host-restriction enzymes (HDR). Broadly, HDRM systems read the pattern of chemical modifications to DNA at host-determined (HD) sites to regulate the fate of incoming mobile DNA. An inappropriate pattern may be restricted either due to the absence of protective modification or its presence; the latter activity is mediated by modification-dependent restriction enzymes (MDRE). Most often, restriction occurs via nuclease-mediated degradation, but it can also act via other mechanisms that prevent the initiation of replication. Like other genome-defense systems, HDRM systems are highly diverse and somewhat modular. The basic functions required for action *in vivo* and the protein domains responsible for each function are addressed here. Particularly under-studied among the latter are the interaction domains that control the launch of highly toxic activities such as HDR. These have been evolutionarily shuffled to build a variety of classical RM systems as well as more divergent systems.

## INTRODUCTION

Understanding the mechanisms that modulate horizontal genetic exchange in prokaryotes is a key problem in biology (1–3). DNA may enter cells via several routes: by infection or transduction carried by virions (bacteriophage or phage); as naked DNA actively imported via natural transformation; or conveyed directly from the donor to the recipient cell by conjugation (4–6). DNA may also be taken up via the incorporation of extracellular vesicles (7).

Host-dependent (HD) restriction-modification (RM) systems (HDRM) are present in most prokaryotic genomes (8). They regulate the fate of entering DNA by reading its pattern of base and backbone modifications. Broadly, RM systems utilize the pattern of chemical modifications to DNA at host-dependent (HD) sites to determine whether incoming mobile DNA should be restricted, most commonly via nuclease-mediated degradation (1–3). Like other defense systems, HDRM systems are highly diverse and modular (9). HDRM systems can be described in terms of the basic functions required for RM *in vivo* and connect these with the protein domains responsible for each function (see “Organization and function of protein domains in classical HDRM Types I-IV”). These domains have been evolutionarily shuffled to build the classical types of RM systems [see “Type IIP (palindromic) systems” and “Type IIS and Type III: asymmetric target sequences”] as well as a variety of more divergent systems (see “New HDRM enzyme classes”). The two core functions of HDRM—DNA modification and restriction—are briefly introduced below.

This review specifically focuses on the biological aspects of HDRM. The very extensive *in vitro* characterization of the enzyme components of Type II RM systems, with very simple cofactor requirements and fixed modification and cleavage positions, was well surveyed in (10), as well as in REBASE (1, 2).

### Modification

Modification enzymes encoded by host cells confer host-dependent modifications (HDM) to resident DNA molecules during replication or maintenance. The great majority of these enzymes are methyltransferases (MTases) that introduce 5-methylcytosine (5mC), N6-methyladenine (6mA), or N4-methylcytosine (4mC) (11) to specific sequence contexts after DNA synthesis. The 7-deazaguanine derivative ADG has also been identified as an HDM associated with the restriction of transformation (12); the associated HDR activity has not been further explored (13). The sugar-phosphate backbone can also be modified with phosphorothioate moieties (14) [see “Phosphorothioate (PT) modification as protection and target”].

**Fig 1:**
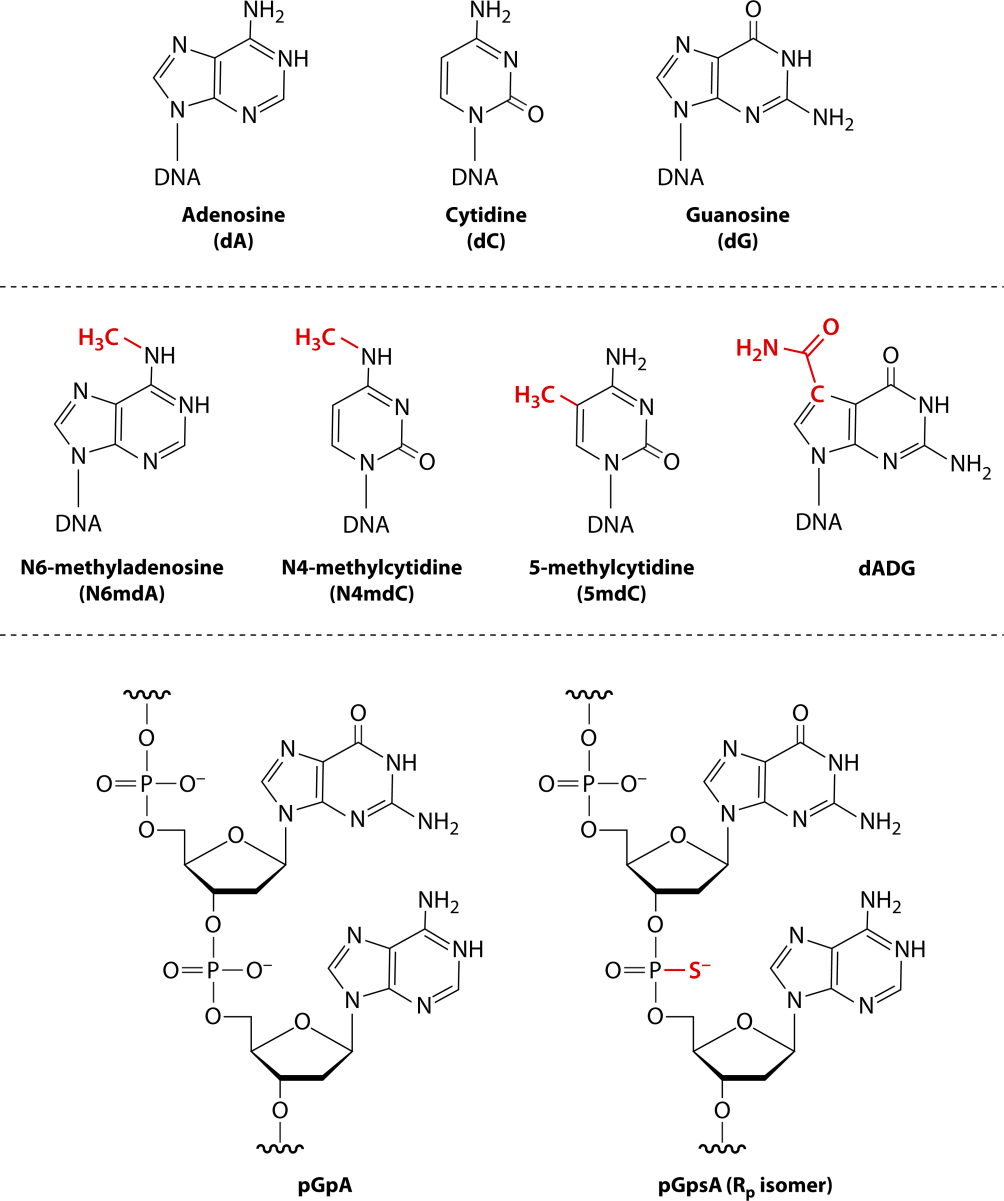
Host-dependent DNA modifications of prokaryotes. Host-dependent nucleobase and backbone modifications are used by cells to distinguish self from non-self. The top row illustrates the canonical nucleobases modified by HDRM systems. The second row illustrates their modified derivatives with the added chemical groups indicated in red. The bottom row shows a canonical phosphodiester backbone (left) and its Rp-phosphorothioate derivative (right) containing sulfur replacing the non-bridging oxygen.

**Fig 2:**
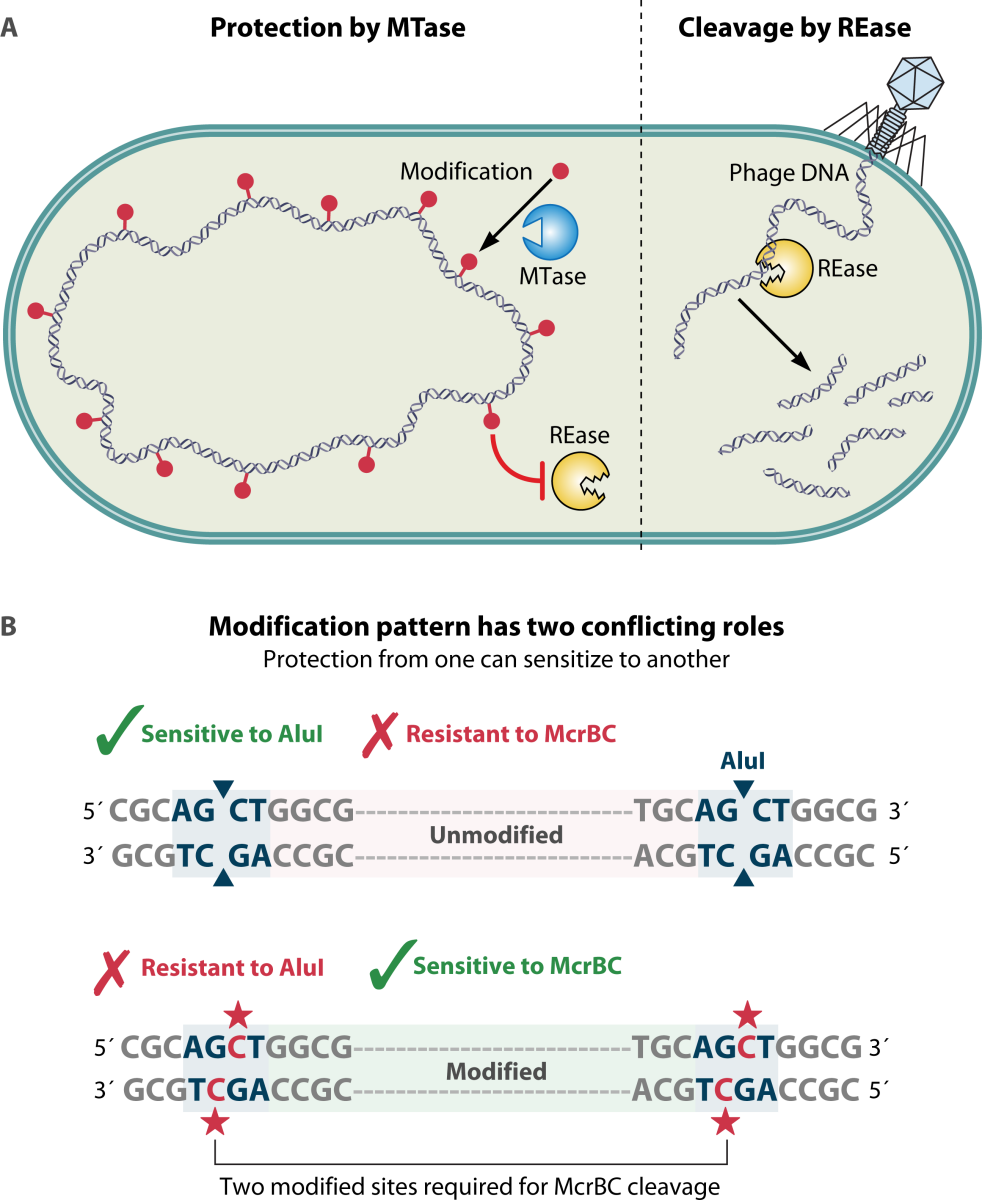
Pattern reading: inherited DNA modification patterns direct HDRM action**.** (**A**) Endogenous chromosomal DNA is modified by an MTase (HDM) and thus protected from the partner REase (HDR), whereas unmodified phage DNA infecting a new host is restricted. (**B**) Modification that protects from one REase may cause sensitivity to another. M.AluI methylates the cytosine of AGCT to protect against R.AluI. Type IV MDRE McrBC cleaves such modified DNA when two RmC are appropriately spaced.

All HDM decorations (Fig. 1) occur within a suitable sequence context defined by the modification enzyme and/or associated proteins. HDMs result in changes to DNA structure that can be read by other DNA-binding proteins, with wide-ranging effects on DNA physiology and gene expression that are independent of the HDRM (11, 15). Many modification enzymes are conserved in particular lineages, but without a partner restriction function (11, 15). These lineage-specific (orphan, solitary) enzymes contribute to the pattern of HDM presence or absence. The whole pattern of orphan and HDRM-associated modification is then interrogated by a new set of HDRM systems upon transfer to another lineage (Fig. 2) (see “Temporal studies of HDRM establishment in new host cells”).

### Restriction

In classical RM systems (Type I–III), the MTase activity is paired with a restriction endonuclease (REase): endogenous DNA methylated by the system’s MTase is protected from restriction, whereas DNA not so methylated is cleaved by the system’s REase and destroyed by the host (Fig. 2A) (1). Typically, these RM systems methylate and restrict at 4–8 base pair motifs that are recognized and discriminated by both the MTase and REase functions. In Type IV systems, modification-dependent restriction enzymes (MDREs) directly attack the modified DNA and are not paired with a modifying enzyme. Thus, REases control DNA entry into a host cell by discriminating the inherited modification pattern of the ancestral host. Indeed, HDRM systems are often incompatible with one another: methylation to protect from one HDRM system may cause lethality in the presence of an MDRE targeting the same modification (16, 17) (Fig. 2B).

Although endonucleolytic DNA cleavage is the cause of DNA restriction in many cases, replication can be blocked without detectable DNA degradation. In BREX systems (18), for example, restriction can be caused by abrogation of DNA replication in response to a foreign methylation pattern (see “BREX type 1: restriction without cleavage”).

### HDRM in prokaryotic evolution and ecology

Host-dependent modification patterns diverge rapidly in populations, typically due to the gain and loss of HDRM systems from genomes (see “HDRM and genome organization and transfer”). In contrast, divergence of overall chromosomal sequence identity is much slower (8, 19). Endogenous diversification of HDRM can result from programmed divergence within a lineage. This can occur by recombinational exchanges of protein cassettes or promoters. The most rapid methods of such divergence, referred to as “phase variation,” establish one or more subpopulations of descendants with a different set of activities (20). This replication slippage or site-specific recombinases are typically involved. When this phase variation affects the expression of a MTase, the resulting changes in HDM patterns can significantly alter transcription throughout the genome; the set of affected genes is sometimes termed a “phasevarion” (21).

Rapid divergence also results from horizontal transfer of HDRM genes themselves (see “HDRM and genome organization and transfer”). The establishment of functional systems in a new host is facilitated by the physical proximity of necessary genes to each other. The genes for HDRM components are often found clustered in genome “defense islands” (22–26). Defense islands usually include HDRM systems that track DNA history (as reflected by the modification pattern), interspersed with other defenses that ignore the modification state. These additional defenses may include CRISPR systems (which read nucleotide sequence independently of modification patterns), abortive infection (ABI), toxin-antitoxin (TA) pairs, nucleases triggered by interaction with phage components (27, 28), and very likely many undiscovered mechanisms.

Regulation of HDRM gene expression and action is a major requirement for success, during replication [see “Type IIP (palindromic) systems,” “Type IIS and Type III: asymmetric target sequences” and “Type I HDRM systems: asymmetric sites and two-strand modification”], as well as during the establishment of newly imported HDRM systems [see “Type IIP (palindromic) systems” and “Temporal studies of HDRM establishment in new host cells”]. Since most prokaryote genomes encode one or more HDRM systems (8), inter-strain transfer of HDRM systems to a naive recipient may encounter a modification pattern incompatible with an incoming system. To establish a new system, the chromosome of an adoptive host must be preserved upon entry and subsequent expression of the new HDR activity. Accordingly, the separate activities of restriction and modification can be regulated to foster horizontal transfer [see “Type IIP (palindromic) systems”]. For multicomponent systems (which are abundant), the kinetics of subunit assembly may protect a naive host (see “Type I HDRM systems: asymmetric sites and two-strand modification”).“

Naturally, phages, plasmids, and other mobile genetic elements (MGE) take advantage of any conserved property of HDRM to devise antirestriction activities (see “Anti-RM activities and host countermeasures”). These include semi-specific orphan methyltransferases (no adjacent REase) that protect only DNA to be exported (29–31) or DNA-mimicking proteins (32). Systems that share a sensitivity to a phage or plasmid countermeasure may also share mechanistic strategies at the enzyme level. Given extensive coevolution between MGEs and their hosts, and the broad prevalence of HDRM systems across prokaryotic taxa, these antirestriction mechanisms appear as diverse as the HDRM machinery they counteract. Furthermore, antirestriction strategies used by MGEs create new vulnerabilities, which may be exploited by the host for defense. For example, elaborate hypermodification of phage DNA creates targets for new MDRE nucleases (33).

There are many additional defense strategies; we refer the reader to recent reviews of: orphan/lineage-specific MTases/epigenome (15, 34); phage hypermodification (33, 35, 36); defense islands (25–27, 37); abortive infection/TA/CBASS (38–41); “adaptive immunity,” CRISPR (42–46); and nucleotide monitoring (47, 48). It is worth emphasizing that these multiple defense systems may act synergistically in some cases (49).

## ORGANIZATION AND FUNCTION OF PROTEIN DOMAINS IN CLASSICAL HDRM TYPES I–IV

**Fig 3:**
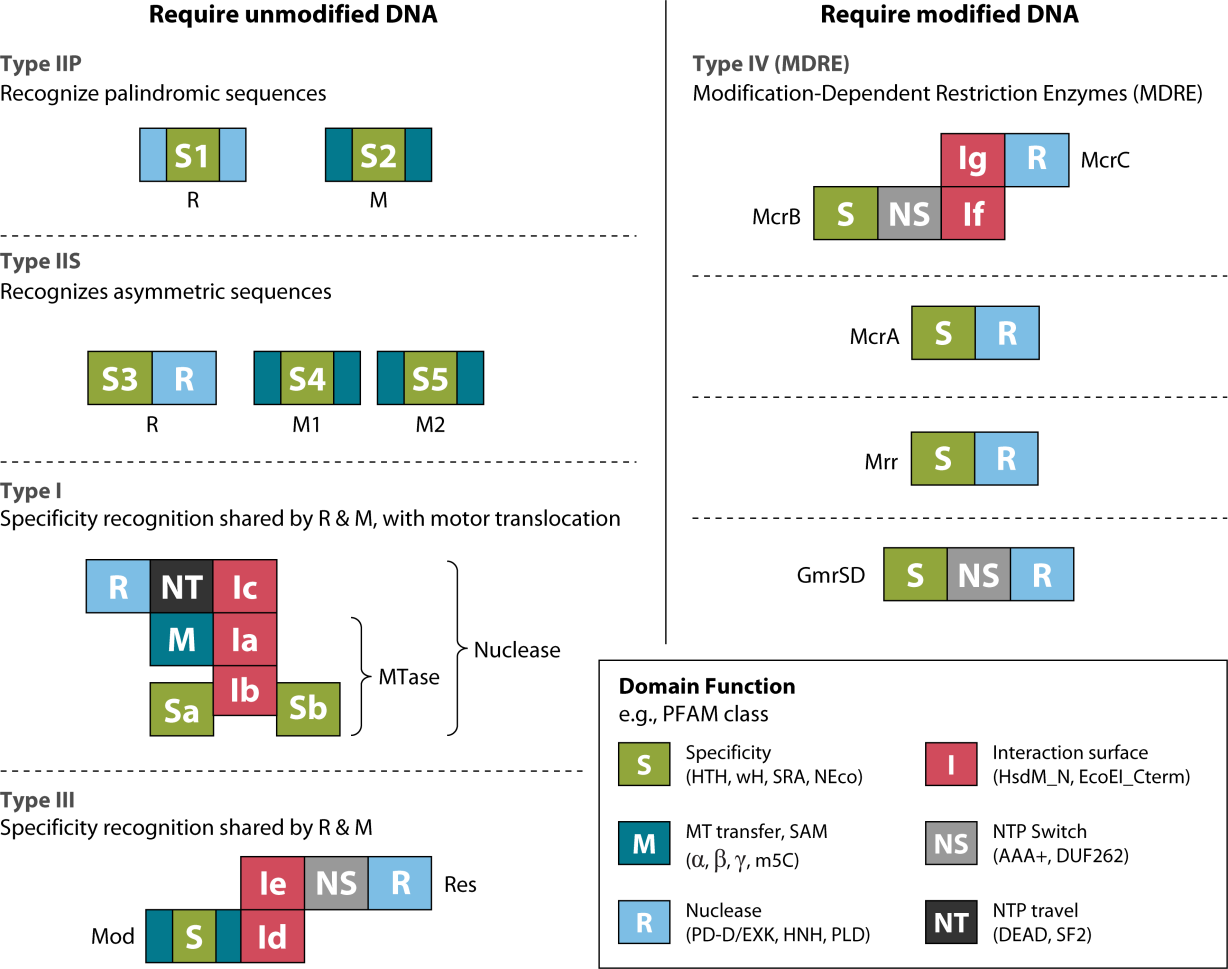
Functions that enable RM and composition of system types. Colored boxes represent protein domains with distinct functions (box on the lower right): site recognition specificity, modification catalysis, DNA cleavage, regulatory protein-protein interaction, and NTPase to facilitate scanning DNA for sites. Functions may be fulfilled in multiple ways. Distinct solutions may be recognized bioinformatically, as by Pfam signatures (Pfam is a database of protein families that includes their annotations and multiple sequence alignments generated using hidden Markov models; non-exhaustive examples in parentheses). Four families in which HDR activity acts on unmodified DNA are shown on the left, whereas another four families shown on the right attack when a sensitizing modification is present: collectively, these are Type IV systems (MDRE). See text for elaboration.

HDRM systems use a variety of protein domains to accomplish their core functions. These functions include the following: selection of DNA sites for modification; modification catalysis; probing the modification state; acting as switches to coordinate other activities; protein complex formation to enable translocation along DNA; and imposing restriction (Fig. 3).

Specificity (S) denotes the recognition of sites in DNA, accomplished by diverse DNA-binding domains. Sites may be 1 bp (e.g., 30) to 8 bp (e.g., 50) long. Modification (M), originally defined in reference to the methylation of a target base by S-adenosylmethionine (SAM)-dependent transferases, has now extended to include phosphorothioate backbone modification as well as the incorporation of a non-canonical 7-deaza-deoxyguanine at a sequence motif. The mechanisms of base methylation vary in detail according to the acceptor on the base; exocyclic amine (i.e., N6 of adenosine or N4 of cytosine [51, 52]) versus C5 of cytosine [53]). Restriction (R) traditionally comprises nucleolytic cleavage or occasionally base excision in response to a site lacking protective modification (54–56). Key coordinating functions are dependent on protein interaction domains (I), allostery or switching regulated by nucleotide hydrolysis (NS), and translocation along the DNA polymer by NTPase-driven motors (NT).

Unlike their more complicated Type I and III counterparts, the majority of Type II systems (tools familiar to molecular biologists) use different S components, each encoded within the polypeptides catalyzing REase and MTase activities. This means that completely different structural solutions have evolved to recognize the same site with the needed fidelity *in vivo*.

## TYPE IIP (PALINDROMIC) SYSTEMS

Type IIP enzymes act on palindromic target sequences (e.g., AluI of Fig. 2B; top left in Fig. 3) (1, 3). In general, these REases act as dimers, with each monomer recognizing a half-site (S1), whereas the MTase recognizes the full sequence of one strand (S2). Due to the symmetry of the site, the MTase substrate is the same on both strands, so that the host can be protected on both strands with a single MTase S domain. It is unclear what evolutionary pressure brings together independent R and M proteins to achieve this result. In any event, coordination of expression upon introduction into a new cell is a particular problem for this category.

### Transcriptional control configurations for Type IIP

We next consider those HDRMs that express the active potentially lethal REase without coupling to the MTase activity. This includes most Type II HDRMs (except Type IIC mentioned above [1]). (For completeness, we note here another subtype, Type IIT, where the REase is independently active but is specified by two genes [yielding a heterodimer where each DNA strand is cleaved by a different subunit] [57–60].) There are thus possibilities for controlling IIT REase activity that is not open to the majority of Type II systems, although to our knowledge, the regulation of Type IIT HDRM genes has not yet been studied. We next consider four distinct regulatory mechanisms for Type II HDRMs with independent REase activity.

#### Regulation by methylation of the promoter

**Fig 4:**
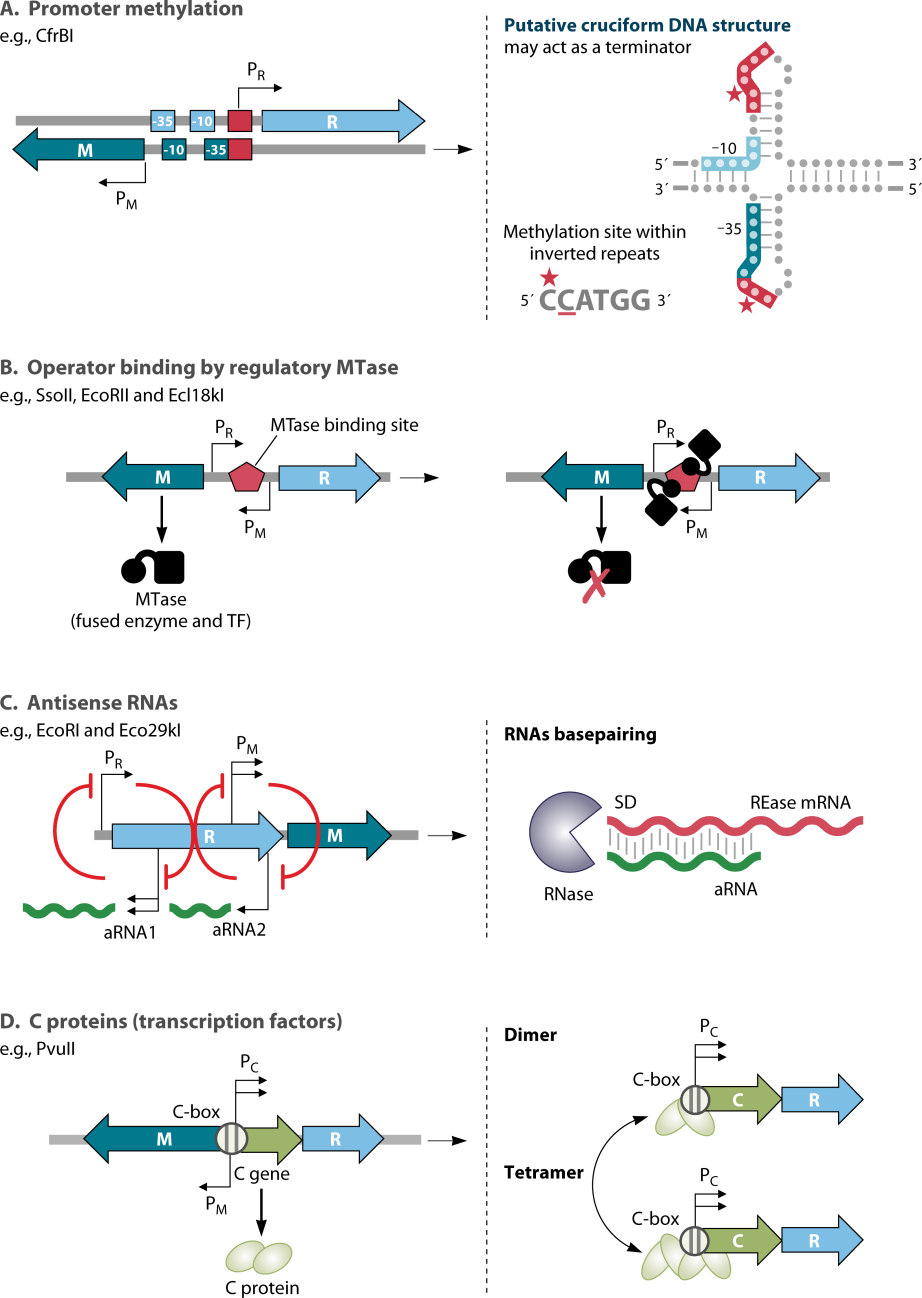
Regulatory mechanisms of Type IIP HDRM. (**A**) Regulation by promoter methylation, where the coordinated expression of an RM system relies on the methylation status of the promoter of the MTase gene (in the case of CfrBI localized within inverted repeats), amid two divergent overlapping promoters for the REase (PR) and MTase (PM). In this system, MTase gene transcription is repressed as the accumulation of methylation in its promoter decreases RNA polymerase (RNAP) binding, allowing more transcription from the competing REase promoter. The putative cruciform structure formation may limit REase expression by terminating transcription from the REase promoter. (**B**) Operator binding by Regulatory MTase. Regulatory MTases are translationally fused to a sequence-specific DNA-binding protein with a helix-turn-helix (HTH) domain at its N-terminus. A binding site for the HTH domain is present between the overlapping REase and MTase promoters. The MTase HTH competes with RNAP for PM binding, leading to MTase auto-repression and increased availability of PR to RNAP. (**C**) Regulatory antisense RNAs are driven from two strong reverse promoters, located within the REase gene. These act by inhibiting both PR and PM, via negative feedback loops. In addition, resulting mRNA/aRNA duplexes are susceptible to RNase degradation, leading to decreased mRNA levels. (**D**) Regulation by dedicated transcription factors. C (control) proteins activate PCR transcription at low C concentrations and repress it at higher levels, via autogenous feedback loops. C proteins bind and distort the DNA operator sequence within its own promoter via HTH motifs, forming homodimers (activators) or tetramers (repressors). Abbreviations: M, modification enzyme gene; R, restriction endonuclease gene; C, control protein; aRNA, antisense RNA; PR, promoter of REase; PM promoter of MTase, PCR, shared promoter of C gene and REase, SD, Shine-Dalgarno sequence.

Requiring both the presence of the protective MTase and its functioning, to transcribe the REase gene, seems like a particularly logical regulatory mechanism—one that is apparently rarely used. In the CfrBI HDRM, the REase and MTase genes are divergent, and their promoters overlap (Fig. 4A). RNA polymerase binding to the two promoters is mutually exclusive. Methylation by CfrBIM of a site in the promoter overlap region selectively blocks RNA polymerase (RNAP) binding to the MTase promoter, thus allowing binding to the REase gene’s promoter (61, 62). In addition, the inverted repeats may generate a cruciform structure that terminates REase transcription, although this has not been tested experimentally. There are substantial numbers of RMSs in which the MTase and REase genes are divergently oriented (63). The AhdI and LlaDII systems are also regulated by promoter methylation, but in this case, only the MTase gene itself is affected (64, 65).

#### Regulation by dual function MTase repressors

Another way to involve the MTase directly in HDRM regulation is to make use of polypeptides in which the MTase is fused to a transcription factor (66–70). Although this approach is, in some cases, insensitive to MTase catalysis, it does at least consistently tie HDRM gene expression to the presence of MTase protein. Ecl18kIM is reminiscent of the AhdI system (see above) (Fig. 4B), in that there is mutually exclusive binding of RNA polymerase to the overlapping MTase and REase promoters. However, in AhdI, the MTase promoter binding is controlled by MTase activity, whereas in Ecl18kI, the MTase promoter access is controlled by the repressor domain of the MTase (66, 68). This is probably true as well for the related SsoII system (70). In the case of EcoRIIM, which also has a fused repressor domain, the REase gene is not affected, but the MTase represses its own gene only when its substrate sites are methylated. This was demonstrated by showing that a catalytically inactive mutant of EcoRIIM still represses, but only if a different, isospecific MTase (Dcm) has modified the EcoRII substrate sites (69).

#### Regulation by antisense transcription

In some HDRMs, translation and/or stability of the REase mRNA is controlled by antisense transcripts originating from within the RMS. The best-studied examples of this are EcoRI and Eco29kI (71–73) (Fig. 4C). (As an aside, the REase of at least one Type III HDRM may also be controlled in this manner [74].) For both EcoRI and Eco29kI the REase gene precedes that of the MTase. The REase promoter drives a long bicistronic mRNA, whereas a separate MTase promoter produces its own transcript, so that MTase mRNA is more abundant than REase mRNA. However, REase transcription is additionally inhibited by tandem, strong reverse promoters. The resulting antisense transcription could have two (untested) effects: favoring MTase production (by decreasing that of the REase) and delaying REase production upon entry to a naive host cell (see “Temporal studies of HDRM establishment in new host cells”).

More specifically, the reverse promoters have two roles. The first role is to inhibit the convergent MTase and/or REase promoters (PR, PM) via transcriptional collision of two RNAP complexes, although the extent to which that happens depends on inter-promoter spacing (75 and references therein). The second role is to produce antisense RNA to base pair with REase mRNA, thus metabolically destabilizing it and blocking the initiation of REase translation (the paired antisense RNA overlaps the Shine-Dalgarno translation initiator). This has been experimentally demonstrated for Eco29kI (72) and many toxin/antitoxin systems (76). This pattern of convergent promoters may be associated with negative feedback loops, potentially acting as a bistable switch (77), which, in theory, could generate cell subpopulations having (respectively) low- and hyper-restrictive phenotypes to neutralize phage infection in the overall population while limiting post-segregational cell killing.

#### Regulation by independent transcription factors (C proteins)

Many HDRMs include genes for transcriptional regulatory proteins that act on the transcription of that HDRM (Fig. 4D). Such autogenous control presents potential advantages (78–80), including the mediation of temporal delays in the expression of REase vs. MTase genes (see below). The genes for regulatory (“controller”) C proteins were first recognized in the PvuII and BamHI Type II HDRMs (81, 82). The C protein family is widespread among Type II HDRMs (83, 84). Interestingly, in some cases, the C protein and REase are fused into a single polypeptide (85), reminiscent of the MTase-repressor fusions described above. A C gene family member is also associated with the Type I *hsd* locus of *Mycoplasma gallisepticum*. It is found upstream of the others (*hsdM*, *S*, *R*) and appears to repress the entire operon (86).

For typical Type II loci, the C proteins bind upstream of and activate their own promoters. This also boosts the expression of the downstream, co-transcribed REase gene. To prevent overexpression from this positive feedback loop, the C proteins also repress their own promoters at higher C concentrations by binding cooperatively to a nearby site that overlaps the promoter (64, 87–89). Specific loss of the repression site depressed the ability to become established in a new host cell (87), due to toxic overexpression of the REase. In some cases, the C protein also represses the MTase gene (88–91). In one reported case, the C protein controls only a portion of the REase gene transcription (92). Even so, deletion of the C gene still leads to higher REase levels and (importantly) a competitive disadvantage relative to cells with the WT Csp231I HDRMs. In another case, which further illustrates C protein regulatory flexibility, the C protein binds only at the MTase promoter, repressing it by RNAP exclusion while allowing access to the overlapping, divergent promoter for the C and REase genes (93). In this arrangement, C has a positive feedback effect on its own transcription via interference with a competing promoter rather than by directly activating its own. This regulation by modulating competing promoters is reminiscent of the control described above for Ecl18kI and SsoII.

Cells in which the C protein has been pre-expressed cannot be transformed by the intact HDRM, in systems such as PvuII (94), due to premature expression of the REase. Related C proteins can cross complement (95), which can lead to mutual exclusion of C-controlled HDRMs resulting from the premature expression of the incoming REase gene, due to promoter activation by the resident C protein (96). The C protein can also interfere with non-RMS regulators, in some cases, with lethal consequences (86, 97, 98). In addition, off-target binding can occur (as with many transcription factors). If this perturbs existing regulons in the new host cell, there should be selective pressure on both the C protein and the host regulatory circuitry to minimize the effect (99–101).

### Possible translational control of Type II and assembly regulation of single-subunit systems

There is evidence for regulation of HDRM gene expression at the translational level, although the mechanisms have not always been characterized (Type II [102]; Type III [103]). The use of antisense RNAs or hairpins to limit access to REase translation start sites has been observed (71, 104). The potential exists for translational coupling between the upstream C gene and downstream REase gene (see Fig. 2 in reference 105). Finally, a cluster of disfavored codons early in the REase gene may affect translation rates (106), although the effects might vary with the host organism (107).

In addition, although the great majority of Type II HDRMs considered above have REases that act independently of their MTases, there are Type II systems for which the MTase and REase activities are connected (1). These include Type IIC, where the two activities are part of a single polypeptide (108–111). Because most of the MTases are active as monomers while REases are typically dimeric (112), the accumulation of Type IIC polypeptide in the cell should theoretically pass through a concentration range within which they remain monomeric (so catalytically M^+^R^-^). Thus, REase activity would be regulated by assembly.

### *In vivo* consequences of unbalanced expression for host and phage survival

Regardless of the HDRM molecular logic used to balance the MTase and REase activities, any disturbance in the MTase-to-REase ratio drastically affects the level of cell protection against phages, ranging from high resistance to the point of cell susceptibility to phage invasion. This occurs due to local stochastic variations in the intracellular amounts of REase and MTase, leading to fluctuations at the single-cell level. When the MTase level temporarily exceeds that of REase, the invading phage DNA may be methylated, allowing the virus to evade restriction and infect the initially protected cell population (113). The MTase to REase ratio can also be affected by the timing of the host cell division, gene copy number, and replication rates, contributing to heterogeneity within bacterial population, as shown by mathematical modeling of HDRM dynamics (114). On the other hand, some HDRMs with the highest restriction efficiency against bacteriophages exhibit also a slight bias toward an excess of REase. This leads to a higher rate of self-restriction, where the host’s own DNA is occasionally cut (115). However, these self-inflicted DNA breaks can be repaired, allowing the cell to survive. Overall, the dynamic balance of HDRM enzyme activities reflects an evolutionary trade-off for the host: maximizing protection against invasive DNA while minimizing damage from self-restriction (116).

Some number of HDRMs located on plasmids contribute to the post-segregational host killing (PSK), thereby enhancing plasmid stability (117, 118). The key feature of PSK-positive HDRMs does not seem to be the enzyme’s cellular lever, but rather their long-lasting activity, which is maintained across cell division cycles to target a significant number of recognition sites. It has been demonstrated that REase activity needs to persist for at least two chromosome replication cycles to effectively kill cells that have lost the HDRM plasmid (119).

## TYPE IIS AND TYPE III: ASYMMETRIC TARGET SEQUENCES

### Type IIS (shifted) systems

For many M-protected families, target sequences are asymmetric. Several architectural solutions are briefly outlined here and illustrated in Fig. 3.

Typically, Type IIS REases recognize asymmetric sites and are modular, with one domain for target recognition and one for cleavage, rather than having a single domain responsible for both activities (as with Type IIP). As a result, because the recognition site is occupied by the S domain, for steric reasons cleavage is “shifted,” that is, occurs outside but nearby to the recognition site itself.

An important problem is now encountered by the modification component of a Type IIS system: how to replicate the asymmetric sites while maintaining protection. Since the two parental strands are not identical, separate M recognition domains are needed to modify both daughters following DNA synthesis.

One solution is to express two distinct MTase enzymes (shown in Fig. 3 as S4 and S5 domains); in that case, both parental strands will be modified. A fusion of two DNA binding domains is sometimes seen (63).

Another general solution to transient loss of modification information during replication is embodied in the R proteins. These may demand inspection of multiple sites before launching restrictions. The adjacency of DNA-bound components is frequently required for efficient cleavage (10, 120–123). Evidence for a multimerization requirement is primarily biochemical, based on rate effects *in vitro* and the common requirement for a “bystander oligonucleotide” (e.g., the PaqCI activator provided with NEB R0745, and discussion of two-site enzymes [see https://www.neb.com/en-us/nebinspired-blog/using-paqci-for-golden-gate-assembly]).

An alternative is to use the same protein component to direct DNA binding by both activities. Sharing of S domains simplifies the job of keeping R and M activities aligned but introduces the need to coordinate the opposing catalytic activities (124)). Much of the variability in RM system composition stems from the need to coordinate recognition by distinct activities. Coordination employs protein interaction surfaces (I), sensing and regulation by NTPase switches, and translocation along the DNA (Fig. 3) (see “Mechanism of activity regulation differs among Type I system families”).

### Type III HDRM systems: asymmetric sites with one-strand modification

**Fig 5:**
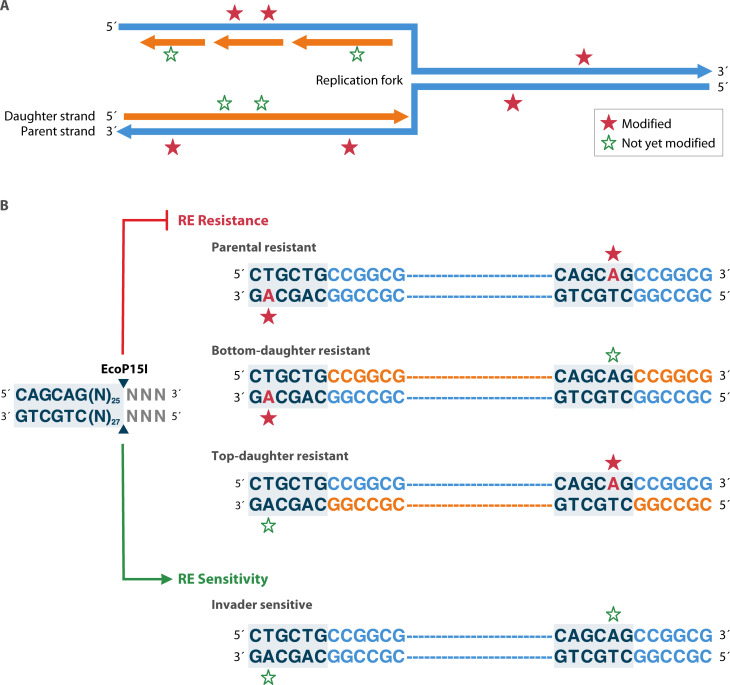
Protection of asymmetric restriction targets during replication. (A) A replication fork moving from left to right lays down a new strand (orange arrows) instructed by the parental strand (blue arrows). Recognition sites for RM action on the parental strand are already modified (red stars), but sites on the daughter strand are not yet modified (green open stars). (B) M.EcoP15I methylates (5’ CAGCAG 3’) on only one strand; restriction requires two unmodified sites on opposite strands. When two successive sites are head to tail, existing modification protects each of the replicated endogenous copies from nuclease action, while invading unmodified DNA is sensitive.

This third approach, best studied in Type III enzymes, demands the presence of, and coordinate detection of, two unmodified sites in opposite orientations before cleavage can occur. The situation at the replication fork is illustrated in Fig. 5A. Red stars indicate modified (thus protected) sites in parental strands; open green stars are unmodified, potentially unprotected positions in daughter strands.

NTP-dependent proofreading by the Type III enzyme EcoPI5I ensures that it acts only when successive CAGCAG sites are both unmethylated and inverted with respect to one another (red stars) (125–131). *In vivo*, this configuration of required sites assures that neither strand of a cleavable substrate is endogenous. The unmodified sites are recognized by the S component of the Mod protein, whereas the Res protein reads the sequence via interaction (“I”) with Mod (Fig. 3). NTP hydrolysis by the Res nucleotide sensing (NS) domain activates the R domain, allowing movement along the DNA: head to tail collision between activated complexes stimulates cleavage near the original site. With this arrangement, all chromosomal sites are already modified on the parental strand, whereas unmodified sites on the daughter strand are all in the same orientation; exogenous sequences are normally sensitive. Escape is possible: phage T7 is not susceptible to restriction by EcoP15I despite having 36 sites (all head-to-tail); the related phage T3 is restricted with at least two pairs of inverted sites (125). This is independent of the anti-restriction functions of these phages (see “Anti-RM activities and host countermeasures”), which do not inhibit Type III enzymes.

Surveillance of this sort may employ powered nucleotide translocation (NT) rather than sliding, as with Type I enzymes and their relatives Type ISP (132, 133). The newly described BREX systems (see “BREX type 1: restriction without cleavage”) also recognize asymmetric sites, modify only one strand, and likely employ translocation to coordinate modification and interruption of replication.

The two genes of the Type III HDRM EcoP15 system are transcribed (at least *in vitro*) from two separate promoters, though there is a limited amount of readthrough from the upstream MTase promoter through the REase gene (134).

## TYPE I HDRM SYSTEMS: ASYMMETRIC SITES AND TWO-STRAND MODIFICATION

Another way to address asymmetric sites is to coordinate recognition of distinct nearby sites on the opposite strands, using linked DNA binding domains (Sa_Sb) assembled with a separate modification domain (M) to carry out methylation, and an additional assembly with a cleavage domain (R) to mediate restriction (Fig. 3). Coordination of the three actions (two modifications and a cleavage) relies on inter-domain interactions and NTP-dependent translocation to detect widely separated unmodified sites (135) [see “Type IIS (shifted) systems”]. Type I enzymes of *Escherichia coli* strains and *Salmonella enterica* serovars were in fact the first HDRM systems to be characterized *in vivo* (136–138), and the first examples of modular DNA recognition (139). *In vivo* recombination among modules can lead to combinatorial variation of Type I HDRM systems (see “Type I protein families defined by subunit exchange”).

### Transcription of Type I HDRM systems

Overall, there is surprisingly little information on the transcriptional regulation of these HDRMs, which may mean that negative results are underreported. In addition, the MTases in these systems are independently active without the participation of the REase, whereas the REase lacks independent activity. Thus, there are more opportunities for proofreading steps in assembly and action, independent of the host transcription apparatus.

What is known of the classic Type I HDRM EcoKI includes transcription from two promoters, one before the REase gene and the second between the REase gene (*hsdR*) and the downstream MTase (*hsdM*) and specificity subunit (*hsdS*) genes, with apparent transcription terminator downstream of the REase gene (140, 141). This arrangement raised the possibility that REase and MTase expression could be independently controlled. The three EcoKI HDRM genes were independently fused to the *lacZ* reporter gene via Mu*dlac* chromosomal insertions (142). Both promoters were weak but functional. More significantly, upon subculturing stationary phase cultures into fresh medium, *hsdMS* transcription began immediately, whereas that of *hsdR* began only after a 1 h delay. The need for such a delay is less stark than when HDRM genes enter a new host cell but could nevertheless be significant when the cell is recovering from the stresses associated with stationary phase (143). EcoCyc (144) indicates that *hsdS* may have a third, independent promoter that uses the RpoE alternative sigma factor (145), which responds to cell envelope stress, although there is as yet no experimental evidence for its role in EcoKI expression.

### Type I protein families defined by subunit exchange

**Fig 6:**
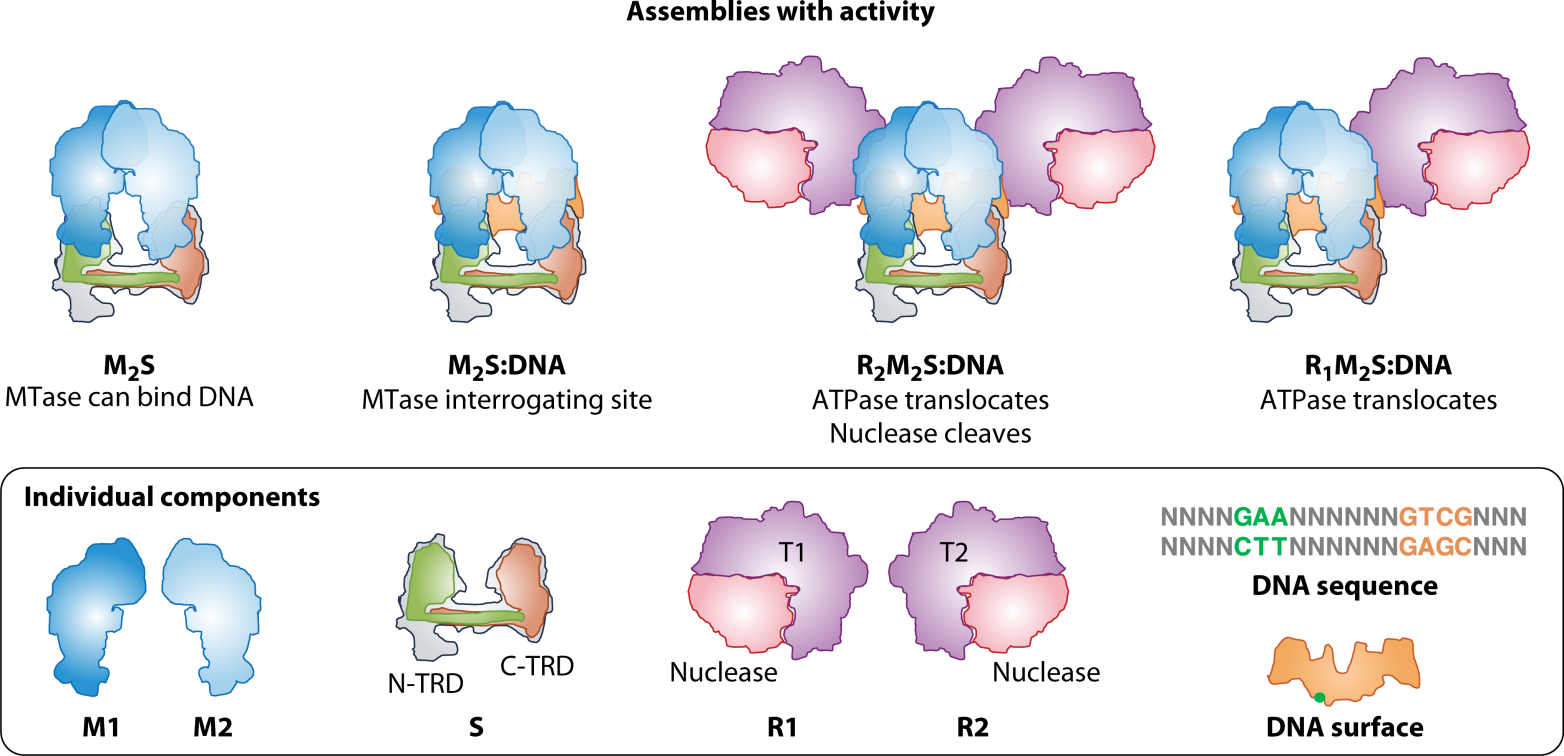
Type I HDRM system protein components, DNA sites, and active assemblies. Top row: protein assemblies competent for different actions; bottom row: individual components that contribute to active assemblies. Components were abstracted from structural visualizations in Fig. 1 and 2 of (146). The complexes there were modeled from crystal structures of EcoKI HsdM (M1, M2), HsdS (S) and DNA, and EcoR124 HsdR (R1, R2). Overall organization and enzyme mechanism are similar. Scale of the M2:S:DNA complex is approximate as presented for M.EcoKI in (146). Type I S proteins exhibit high variability in the Target Recognition Domains (TRD; green and orange blobs), which recognize distinct DNA sequences (green and orange highlights on orange helix). TRDs are connected by conserved helical regions (green and orange bars). Sites comprise 7-8 specific nucleotides (green and orange dots on the DNA structural visualization), with nonspecific spacer. Protection is conferred by methylation of adenine (bold green and orange in the sequence). Catalytic components of HsdR include the nuclease domain (pink region) and two translocase domains (T1 and T2; violet region). Relative scale as between the two figures of (146) has been roughly adjusted to fit the figure here, recalling that HsdR of EcoR124I is 1033 aa, whereas HsdM of EcoKI is 529 aa. Above: Active assemblies. M2S1 is an active methyltransferase while R2M2S1 can either modify a hemi-methylated site or engage DNA to translocate and cleave. For both EcoKI and EcoR124I, an unmodified recognition site triggers translocation (not shown) of flanking DNA by HsdR/T, with R2M2S remaining fixed at the unmodified site. EcoR124I translocates both as R2M2S1 and R1M2S1. R.EcoKI has a required, protease-sensitive N-terminal extension not found on R.EcoR124I. DNA binding by S proteins requires M2. Not shown are cofactors S-adenosylmethionine (SAM), Mg^++^ (both required for all activities), and ATP (required for translocation and cleavage).

Type I components combine to form a molecular machine of five subunits (R_2_M_2_S) that binds to DNA, identifies a recognition site, and assesses the modification state (Fig. 6) before acting as follows. If the site is already fully methylated, the complex dissociates. If hemi-methylated, MTase activity is stimulated, methylation of the unmodified strand occurs, and the complex dissociates from the DNA. If the site is unmethylated, R_2_M_2_S remains bound at the site, whereas the R subunit engages adjacent DNA. R-mediated ATP-driven DNA translocation ensues until another translocating complex or other barrier is encountered, stimulating DNA cleavage. The three-subunit M_2_S assembly can act to modify DNA in the absence of R subunits, but R has no activity independent of the fully assembled R_2_M_2_S complex. The logic is that hemi-methylated DNA is native and newly replicated, whereas incoming DNA should be unmethylated on both strands.

Subunit assembly steps and conformational state transitions restrain lethal action during replication, recombinational repair (147), and even horizontal gene transfer to a naive host. However, details of these restraints differ between Type I families ((148) and see below).

#### Type I exchange families correlate with the separation of M sites

Type I enzyme families were defined based on the phenotypic consequences of this decision-making. The exchange of subunits (especially HsdS proteins) *in vivo* to reconstitute enzymatic activity is the hallmark of each family. This ability depends on the compatibility of the interaction surfaces of different components. A striking correlate of the family structure is the distance between nucleotides targeted for methylation (Table 1). This distance is measured in space by the conserved helical regions (CR) that separate target recognition domains (TRDs) in HsdS. That distance is then communicated to HsdM (Fig. 6, S subunit domain organization TRD1-CR1-TRD2-CR2, where TRD1 and TRD2 recognize two short DNA sequences separated by 5–9 bp). This provides an opportunity to monitor the modification state of both strands in the decision-making process.

**TABLE 1:** Type I families defined by subunit exchange

Family	Enzyme	Recognition sequence^*a*^	D (bp)^*b*^	References^*c*^
IA	EcoBI	TG**A**(N8)**T**GCT	8	(149)
	EcoKI	A**A**C(N6)G**T**GC	8	(149–151)
	EcoDI	TT**A**(N7)G**T**CY	8	(149, 151, 152)
	StyLT7III (StySB)^*d*^	G**A**G(N6)R**T**AYG	8	(149, 153–156)
	StySPI	A**A**C(N6)G**T**RC	8	(149, 154)
	StySQ1	A**A**C(N6)R**T**AYG	8	(149, 154, 155)
IB	EcoAI	G**A**G(N7)G**T**CA	9	(149, 153, 157, 158)
	EcoEI	G**A**G(N7)A**T**GC	9	(157, 159)
	CfrAI	GC**A**(N8)G**T**GG	9	(160)
	StySKI	CG**A**T(N7)G**T**TA	9	(158)
	EcoAIO83I	GG**A**(N8)A**T**GC	9	(161)
IC	EcoRI24I	GA**A**(N6)R**T**CG	7	(162–164)
	EcoRI24I∆	GA**A**(N7)**T**TC	7	(165)
	EcoRI24II^*c*^	GA**A**(N7)R**T**CG	8	(164)
	EcoDXXI^*c*^	TC**A**(N7)R**T**TC	8	(166)
	EcoDXXI∆^*c*^	TC**A**(N8)**T**GA	8	(167, 168)
	EcoPrrI^*c*^	CC**A**(N7)R**T**GC	8	(169)
ID	StySBLI	CG**A**(N6)**T**ACC	6	(156, 170)
	EcoR9I	AGC**A**(N6)**T**GA	6	(156)
	KpnAI	GGC**A**(N6)**T**TC	6	(171)
IE	KpnB1	CAA**A**(N6)R**T**CA	7	(172)

^*a*^ N, any nucleotide; R, either purine; Y, either pyrimidine. Underlined bold type identifies either the adenine that is the target for methylation or the thymine complementary to the target adenine. For EcoEI, CfrAI, and StySBLI, these adenine residues are the sole modifiable bases within target sequences identified using the restriction activity.

^*b*^ Number of base pairs between target adenines.

^*c*^ These Type IC members have four more amino acids than EcoRI24I within the central conserved region, the region that links the TRDs.

^*d*^ Additionally, this table corrects misattribution of the REBASE name of the *Salmonella* MTase previously known as StySB; it is StyLT7III, not StyLTII as in references 173–175] or SenLT7III in REBASE (at the time of this writing). Present numbering adopts the convention of references 176, 177.

### Mechanism of activity regulation differs among Type I system families

Multiple components are involved in decisions: bind DNA; bind SAM; transfer -CH_3_; bind NTP; release NTP; hydrolyze NTP; change conformation; and hydrolyze a phosphodiester bond. Communication between components to make such decisions becomes a new “activity” (Fig. 3, Interaction). Such interaction capacities must coevolve to diverge from an ancestral state.

The different families may be distinguished by the regulatory properties of the subunit interactions. Interacting regions determine which activity of the R_2_M_2_S complex is deployed when a DNA site is detected—modification or restriction. Mutational analysis has identified interaction surfaces that are required for effective restriction. Three distinct antirestriction mechanisms (RA) reduce the barrier posed by RM systems. Effective modification enables transfer to a new host, survival of DNA damage, or survival of phages on entry.

#### Phage-mediated restriction alleviation interferes with maintenance methylation

The first sort of restriction alleviation (RA) suppresses an enhanced self-recognition property called maintenance methylation. Type IA RM systems modify unmethylated sites much more slowly than hemi-methylated sites. Thus, at a replication fork, the parental modification state is transmitted to the daughters efficiently, while reducing successful phage invasion. To counter this property, phage λ employs Ral (67 amino acids) to speed up the modification of the first strand in unmodified substrates (178), resulting in increased phage survival (179). Type IB enzyme EcoA and Type IC EcoR124I do not show this Ral-responsive property (180, 181)

Both the maintenance methylation preference and the sensitivity to phage λ Ral reside in the N-terminal domain of HsdM (~150 amino acids), where mutations that accelerate modification of the first strand were found (182). Significantly, five of the 14 mutations with accelerated *de novo* methylation also lost restriction capacity, implying that the decision to methylate is integrated with the decision to launch restriction. This property is embodied in a well-folded N-terminal segment identified in EcoK HsdM by mutation and partial proteolysis (182, 183). Mutations that destroy preference for hemi-modified DNA sites affect contacts between the two M subunits of the active MTase or folded surfaces within one domain (184).

#### Proteolytic suppression of restriction on entry and in response to damage

Two distinct mechanisms have been identified for restriction alleviation (RA) following DNA damage [designated UV-RA or 2-AP (2-aminopurine) RA depending on the damage]. The type of damage RA goes together with the suppression of restriction activity upon transfer to new hosts (185). Types IA and IB share a mechanism, whereas Type IC exhibits a different one.

During damage repair or upon entry of a Type IA or IB system into a cell while the host chromosome is not yet protected by fully modified sites, the quality-control protease ClpXP degrades the nuclease component HsdR specifically, affecting a delay in R activity, allowing for the establishment of new host modification patterns (181, 186–188). This results both in increased survival of entering phage and increased ease of establishment in a new host. This *in vivo* RA follows 2AP treatment. It does not occur when *clpX* is deleted. In the presence of the drug, EcoK cleavage occurs when unmodified EcoKI sites are created after replication. Chromosomal degradation by RecBCD (Exonuclease V) acting at these double-stranded breaks ensues unless rescued by recombination (189). *In vitro*, ClpX degrades EcoKI only at the critical moment, when fully assembled on DNA, with the ATPase intact and translocation-competent; DNA cleavage is not required to create the ClpX protease substrate from EcoKI (190).

#### Restriction alleviation by assembly failure

Type IC systems also exhibit 2AP-RA and efficient transfer into a naive cell, but the mechanism is distinct (191). In fact, it seems to result from (evolutionary) tuning of enzyme assembly processes. Unlike Type IA enzyme EcoKI, which assembles HsdR onto its M_2_S platform to make R_2_M_2_S, and then binds to DNA, the Type IC enzyme EcoR124I assembles through a cleavage-incompetent R_1_M_2_S:DNA intermediate (Fig. 6) and then adds the second R to enable cleavage. During translocation, disassembly and reassembly occur. EcoR124I differs from EcoKI in that an *hsdR* mutation that reduces its 2AP-RA does not alter transmission efficiency to new hosts (unlike EcoKI); its modification activity does not prefer hemi-methylated substrates. A mutation in the C-terminal region of HsdR of EcoR124 suppresses RA on phage infection (181). This mutation alters the cycling efficiency with which the holoenzyme converts from a cleavage-incompetent R_1_M_2_S_1_ complex to a cleavage-competent R_2_M_2_S_1_ complex (192). This phenotype enhances the dependence of the cell on homologous recombination for survival during episodes of DNA damage (193).

## TYPE IV SYSTEMS: ACTION WHEN SITES ARE MODIFIED

### Introduction to MDRE

Modular organization is also typical of Type IV RM systems, also called MDRE (right side of Fig. 3). Examples include McrBC (22), McrA (194), and *in vitro* for McrBC (195–198) and Mrr-family member MspJI (199). Both modification-protected and modification-dependent systems may employ translocation in the process of detecting and acting on DNA (200, 201). Some functions are emergent, resulting from the interaction between two domains or between the protein and the substrate. Potentially, target recognition (S) is carried out at the interface of some two-domain MDREs, for example, GmrSD homolog BrxU (202).

Modularity of these activities also allows the modification-protected category to exchange components with MDRE systems. For example, some McrBC “homologs” use S domains specific for 6mA instead of 5mC (203). One McrBC-related example even acts on an *unmodified* site when accompanied by suitable MTases (204, 205) (see below).

As DNA sequence and protein-structure databases have expanded, bioinformatic prediction informed by these characterized examples has enabled motif and domain databases to be established, enabling the present discussion. The prediction of novel systems has, in some cases, led to the characterization of new HDRM components (206–208). Exploiting this modularity bioinformatically has also enabled the identification of novel configurations of recognition and cleavage mechanisms (209, 210).

Genetic approaches revealed the existence of MDREs, enzymes that restrict DNA by virtue of the presence of a modification rather than due to its absence. An early example was McrBC of *E. coli* strains K-12 and B (211). This enzyme targets the motif RmC *in vivo*, where mC can be 5mC, 5hmC, or 4mC. All modes of mC-modified DNA entry are restricted, including infection by phage, conjugal DNA transfer, and plasmid transformation. Many patterns of mC modification are targeted by McrBC, including those established in plants and animals (16, 212), as well as those established by cloned genes of many prokaryotic MTases.

MDREs are mechanistically diverse. Recognition of modified bases in DNA may employ domains that otherwise function in RNA or small-molecule metabolism. Mining of rapidly expanding sequence and domain databases has resulted in the identification of new such domains, which are typically attached to a variety of DNA restriction modules. As with other types of REases, the most common mode of restriction is DNA cleavage, but recently, other mechanisms of DNA damage such as base excision by DNA glycosylase action and sulfur (phosphorothioate) binding to the target recognition sites have been identified.

### Detecting modification: adoption of PUA domains for MDREs

**Fig 7:**
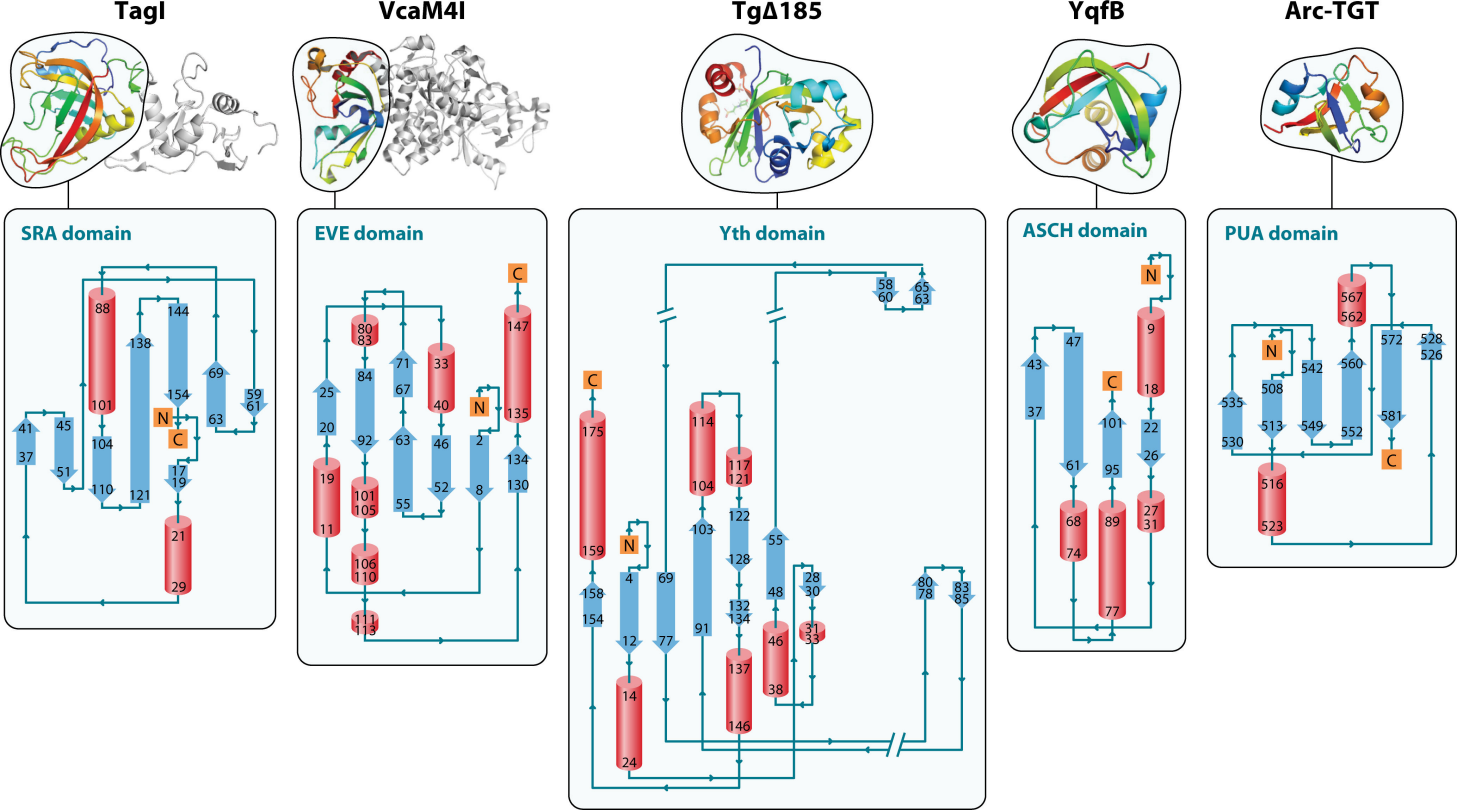
Structure and topology of PUA and PUA-like domains. Top row: cartoon domains rendered in rainbow colors (N red- to-C violet); bottom row: associated domain topology renderings. Left to right: 5mC-restricting TagI endonuclease residues 1-164. TagI PDB: 6GHS.ee, with SRA domain topology; VcaM4I endonuclease restricts 5hmC and 5mC (rainbow, residues 1-147) PDB: 6YEX, with EVE domain topology: Yth domain from Tg∆185, a naturally-occurring Yth-McrB fusion that binds to 6mA modified DNA. PDB: 6P0F, with Yth domain topology. *E. coli* protein YqfB, an N4-acetylcytidine amidohydrolase that hydrolyzes various N4-acylated cytosines (4acC) and cytidines. YqfB PDB: 1TE7, with ASCH topology; Archaeosine tRNA-guanine transglycosylase (Arc-TGT). PDB: 1J2B. The PUA domain consists of 67–94 amino acid residues with 4–7 β-strands architecture and a few α–helix insertions. In this example, the PUA fold is composed of six β-strands coiled to form a pseudobarrel, also known as a folded β-sandwich with PUA domain topology.

One of the largest families of nucleobase-recognition domains is the PUA family (pseudouridine synthase and archaeosine transglycosylase) (Fig. 7; structural models were visualized and rendered with PyMOL [PyMOL Molecular Graphics System, Version 3.0.4 Schrödinger, LLC] using coordinates downloaded from the Protein Data Bank [PDB]; topology diagrams created with PDBSum [213]). The original family included RNA metabolic enzymes found in a wide range of archaeal, bacterial, and eukaryotic proteins (214). The PUA superfamily consists of a pseudo-barrel structure composed of mixed folded sheets of 5–6 strands that include PUA-like SET, ASCH, EVE, and YTH domains (215–217). PUA family proteins containing the EVE domain were first predicted to recognize modified bases by Aravind and Koonin (218), and more recent bioinformatic analysis by this group has vastly expanded the EVE family (215), which now includes VcaM4I endonuclease (219). YqfB, an ASCH-containing protein, hydrolyzes a variety of N-modified cytosine residues (220, 221).

### McrBC family: AAA^+^ NTPase collaboration with NTPase

McrBC base specificity is determined by the N-terminal domain of McrB, McrB-N (195), an N-terminal segment of McrB that is a base-flipping member of the SRA family (222), whereas cleavage is carried out by McrC, a PD-(D/E)XK nuclease (198). Coordination between McrB-N and McrC is mediated by the C-terminal domain of McrB, a tunnel-forming GTPase of the AAA+ protein family (196). *In vitro*, sites with suitable spacing (123) are cleaved in a GTP-dependent fashion following translocation by a complex comprising a McrB(6):McrC (1, 200). The proper assembly enables binding by McrC to stimulate GTP hydrolysis and thus translocation (223).

#### Diversity of DNA-recognition components in the McrBC family

Domain-mining using the McrB GTPase as seed has generated the prediction of a large number of derived systems, including some predicted to target RNA rather than DNA (224). Among those already characterized *in vivo* or *in vitro,* related systems in other organisms retain the overall functional organization of McrBC but employ YTH or EVE in place of the SRA domain McrB-N for base recognition (203, 225). Some GTPase-tunnel-forming relatives even recognize unmodified DNA: the LlaJI RM system recognizes an unmodified, asymmetric site via a B3 DNA-binding domain. LlaJI includes two MTases for full modification of the asymmetric site and viability of its host, and it restricts DNA via the interaction of an McrB-related NTPase with an McrC-related nuclease (204, 205).

#### Activity regulation by the assembly of McrBC

*In vivo* action by McrBC has shown activity regulation by an assembly pathway. In the wild-type *mcrB* gene, internal translation initiation yielding a C-terminal AAA+ GTPase domain is favored, yielding McrB_S_ (226), lacking the McrB-N DNA binding domain. Overexpression of this component withdraws McrC from assembly with the active complex, McrB_L_(6):McrC (196, 227). *In vivo,* this suppresses the restriction of T4*gt* by five orders of magnitude (226). McrBC protein, although susceptible to the protease ClpAP, does not appear to be subject to UV-induced RA using T4*gt* (228) but is subject when assessed with phage λ (229); in neither case were the transcripts measured.

### Two-domain SRA-nuclease fusion MDREs

The MspJI family REases carry an N-terminal SRA-like domain and a C-terminal Mrr-like catalytic domain with a PD-QxK catalytic site (199, 230). No *in vivo* action on phage or plasmids has been explored for the MspJI family enzymes. *In vitro*, MspJI forms a tetramer and cleaves 5mCNNR sites 12 and 16 bases downstream of the modified cytosine, leaving a 4-base 5’ overhang. Cleavage of a fully modified CpG methylated site generates a small fragment of 32 bp with the modified sites in the middle. Such fragments can be purified and sequenced by NGS, which forms the foundation for MspJI-based modified site mapping (scMspJI-seq for single-cell methylome study [231] and MFRE-seq [232]). The recent addition of PsuGI endonuclease (BB5mCGD, B = C/G/T, D = A/G/T) to the MspJI family of enzymes may find additional practical applications in the mapping of modified CpG sites in mammalian genomes

The plasmid-borne Type IV restriction system PvuRts1I has an N-terminal PD-(D/E)XK cleavage domain, and a C-terminal SRA domain for site recognition (233). The activity was discovered due to its restriction activity against wild-type, glucosylated phage T4 (234); see “Occlusion of restriction sites” for more detail on the phage side of the story. *In vitro*, the enzyme restricts both 5hmC and 5ghmC-modified DNA (ghmC = glucosylated hmC). When modified sites are suitably spaced, collaborative cleavage between modified positions can occur. For PvuRts1I, this motif can be described as 5'−5(g)hmC)-N(21-22)-G3' paired with 3'−5(g)hmC-N(21-22)5'; cleavage occurs in the middle of this motif (235, 236). Recognition is mediated by a C-terminal SRA-related domain (233). The cuts sites mediated by the N-terminal PD-(D/E)XK domain appear to be variable (“wobble cuts”: 5hmC-N12/N10-G, 5hmC-N12/N9-G, and 5hmC-N11/N9-G). PvuRts1I and its homolog AbaSI both exhibit the following preference for cleavage of modified cytosines: 5ghmC >5 hmC >>5 mC (236).

An HNH cleavage domain may substitute for the PD-(D/E)XK-related domain. TagI (237) and VcaMI (219) contain an SRA (TagI) or EVE (VcaMI) domain at the N-terminus, with a C-terminal HNH nuclease domain (ββα-metal fold). This forms a dimer in the crystal structure. In heterologous expression of TagI *in vivo*, hmC-containing phage T4gt was strongly restricted, but glucosylated T4 was resistant. *In vitro*, TagI cleaves both 5mC and 5hmC-modified DNA. It cuts DNA modified by M.Fnu4HI (G5mCNGC), Dcm methylase (C5mCWGG), and M.HpyCH4IV (A5mCGT), suggesting a very relaxed recognition sequence.

A variation of the two domain SRA-nuclease is SauUSI endonuclease from *Staphylococcus aureus* USA300 that contains N-terminus PLD nuclease domain, ATPase (helicase) domain, and C-terminus SRA domain. This enzyme requires ATP or dATP for activity and cleaves near the modified sites 5′-S5(h)mCNGS-3′. SauUSI homologs can be found in many sequenced *S. aureus, Staphylococcus epidermidis,* and *Staphylococcus carnosus* genomes (2, 238).

### GmrSD family MDREs: DUF262+DUF1524

Examples include GmrS/GmrD (acting in complex) or GmrSD fusion proteins, which act on DNA-containing modified cytosines (5hmC or 5ghmC). DUF1524 includes a conserved nuclease motif EHxxP typically found in proteins within the His-Metal nuclease family (ββα-metal fold).

The phage-host coevolution spiral (see “Anti-RM activities and host countermeasures”) was illustrated with the identification of T4-encoded inhibitors directed to the GmrSD activity. Small inhibitor proteins (IPIs) encoded by the phage are packaged into phage particles. When delivered with the DNA, they specifically block the action of the GmrSD restriction system on the glucosylated genome with 5ghmC DNA. Specifically, GmrSD *in vivo* restricts T4 IPI-deficient phages by a million-fold (239, 240). Expression of GmrSD in a heterologous *E. coli* host resulted in two proteins, GmrS and GmrD (a stop codon mutation was introduced to generate GmrS, and re-initiation of the second open reading frame yielded GmrD). The complex digested 5ghmC modified DNA in the presence of NTP. A single-polypeptide GmrSD cleaves 5hmC- and 5ghmC-modified DNA in the presence of NTP or dNTP. Bioinformatic analysis of GmrSD homologs in many sequenced bacterial genomes (208) showed that many GmrSD homologs occur that are fused to other protein domains, some of which are classified as DUF (domain of unknown function); potentially, these have evolved for restriction of other modification states.

GmrSD homologs further vary in substrate preferences: BrxU is a GmrSD homolog embedded in a plasmid-borne defense island from a strain of *Escherichia fergusoni. In vivo*, BrxU acts independently of the accompanying BREX system (see “BREX type 1: restriction without cleavage”) to restrict a variety of phages bearing modified cytosines. As with *E. coli* GmrSD, BrxU endonuclease activity was stimulated by an NTP or dNTP. Unlike the protype GmrSD (acting only on glucosylated sites), BrxU displays *in vitro* restriction activity toward m5C-modified DNA, as well as 5ghmC DNA. Structurally, BrxU forms an interlocked dimer in the crystal structure with the N-terminal domain (DUF262) forming the nucleotide binding fold and the C-terminal domain forming the HNH endonuclease fold. The catalytic residues were proposed to be DH-N-N-D (D = Asp, H = His, *N* = Asn) (202).

In the immigration control region of some *E. coli* genomes, the McrBC locus was replaced by a GmrSD homolog, Eco94GmrSD (241) (see “Defense islands: laboratories of evolution and exchange”)

### Winged-helix (wH) domains in MDRE endonucleases

The archetypal Mrr endonuclease of *E. coli* K-12, EcoKMrr, comprises an N-terminal binding domain (winged helix, wH) fused to a Mrr-cat nuclease domain with a PD-QxK catalytic site. Specificity *in viv*o was determined from the reduced entry of MTase expression plasmids and premodified phage into strains with a wild-type Mrr (229, 242–244). Both 6mA and 5mC confer sensitivity in different contexts.

Although no *in vitro* activity has been described for *EcoK*Mrr, extensive *in vivo* studies with expression reporters and single-cell approaches suggest that action is regulated by oligomeric state (245). DNA damage is inferred from *mrr-*dependent induction of SOS-dependent phenotypes *in vivo*, including *recA* transcription (246), filamentation, and nucleoid condensation (247). These are induced by the expression of a sensitizing MTase, M.HhaII (modified site G6mANTC), or by high hydrostatic pressure. The responses to the two stimuli are genetically separable, but mutants that inactivate both responses can be isolated (248). Although high hydrostatic pressure is not a condition to which *E. coli* is typically subjected, overlap may occur between that challenge and other stresses including temperature shocks and oxidative stress (249–252). Neither the EcoCyc (144) nor RegulonDB (253) databases reveal control of *mrr* by transcription factors. Post-translational regulation by oligomeric state has been studied using a fusion of Mrr protein to GFP in *E. coli* cells. Fluorescence microscopy showed that GFP-Mrr was tetrameric in uninduced cells, but the activated GFP-Mrr fusion existed as dimers (254). A model for Mrr activation by methylated DNA has been proposed (245). In this model, the oligomeric state is an activity switch: inactive tetramers are converted to active dimers; these bind tightly to methylated sites and generate incisions nearby. Similarly, for the pressure-induced Mrr activation and SOS response, the HP shock pushes the tetramer-dimer equilibrium toward active dimers, causing incisions in the unmodified DNA. In both cases, after DNA cleavage or nicking, the Mrr endonuclease retains its dimeric conformation and remains irreversibly bound to DNA, localizing to foci associated with DNA damage and nucleoid condensation upon induction of the SOS response.

The endonuclease DpnI, which cleaves fully modified G6mATC sites, comprises an N-terminal PD-ExK catalytic domain and a C-terminal winged-helix (wH) domain that recognizes N6mA residues (255). Both domains are involved in N6mA sensing, with a double-check mechanism in the catalytic process. DpnI has been widely used in molecular cloning and site-directed mutagenesis to destroy methylated host DNA, phage DNA, or Dam-modified plasmid DNA. Hemi-methylated GATC sites are poor substrates (256; see also the REBASE table of modification sensitivity at https://rebase.neb.com/cgi-bin/msget?DpnI).

The effect of the DpnI/DpnII/DpnIII locus on gene flow in the native population of *Streptococcu*s has been extensively studied (257). Most isolates carry one of three alternative gene cassettes with enzymes recognizing GATC. DpnI cleaves fully 6mA-modified GATC; DpnII cleaves unmethylated GATC and is blocked by 6mA; and DpnIII (Spn23FI in REBASE) cleaves unmethylated GATC but is blocked by 5mC. The DpnI cassette would also contribute protection from phylogenetically distant sources, since the broadly distributed Dam methyltransferase modifies G6mATC to regulate DNA replication and mismatch repair parental strand marking (258, 259). Numerous bacteriophages also encode Dam methylase homologs (260).

Designed domain swapping has enabled restriction activity mediated by different nuclease domains: DpnI Wh domain can be fused to HNH and GIY-YIG endonuclease domains and the fusion enzymes restrict N6mA modified DNA by 2–3 log in plasmid DNA transformation (9).

### Additional DNA-binding domains in Type IV REs

McrA of *E. coli* K-12 targets phage infection or plasmid transformation with DNA containing C5mCGG *in vivo* (194). The gene is located on the mobile prophage-like element e14 (261). The protein comprises an N-terminal DNA recognition element (262) that binds modified DNA tightly on its own, and a C-terminal HNH endonuclease domain. The full protein binds as a dimer with weak modification dependence, cleaving in an Mn2^+^-dependent fashion (263, 264). The weak modification dependence of cleavage *in vitro* contrasts with the high specificity of *in vivo* restriction (244). *In vivo*, truncations lacking the endonuclease domain still partially restrict M.HpaII-methylated phage λ 10-fold to 1,000-fold (194) but lack activity on hmC-containing T4*gt*. This could mean that the cell has additional controls on site recognition not recapitulated *in vitro*. Alternatively, since the two phages have very different dependences on host functions, particularly replication factors, restriction of phage λ might be the effect of a different mechanism of interference with phage replication.

In *E. coli* B, both recognition and cleavage are carried out by EcoBLMcrX, a short 143 aa protein specific for 5mC. Two reports gave similar recognition sites: G5mCNGC (as EcoBLI [265]) or the more relaxed sequence R5mCSRC (EcoBLMcrX [266]). This activity is expected to cleave 5hmC in phage T6 as well. The original name for the locus was *E. coli* B *rglA* (gene mnemonic *r*estricts *gl*ucoseless phage). As expected from the original genetic analysis (261), the *mcrX* gene is near the *his* operon (~100 kb away) and is unrelated to EcoK *mcrA*.

### Type IV enzymes acting on phosphorothioate (PT)-modified DNA

With exploration of the novel PT-protected HDRM systems [see “Phosphorothioate (PT) modification as protection and target”], it naturally has emerged that some MDREs target biological modification (267). Such new systems also exhibit modular architecture (210). These have been useful in exploring the sequence preferences of the PT modification in reference 268.

## NEW HDRM ENZYME CLASSES

### BREX type 1: restriction without cleavage

Like the Type I HDRM systems, the recently described BREX systems carefully orchestrate the constituent activities of sequence recognition, cofactor binding, DNA methylation, NTPase action, apparent motor translocation, and replication arrest, but their *in vitro* activity is at present poorly understood. Unlike Type I HDRM systems, no DNA cleavage has been found *in vivo* or *in vitro*. Instead, the infecting phage failed to initiate replication (see below).

The BREX systems were defined bioinformatically by Goldfarb (18) as conserved gene clusters carrying homologs of *pglZ*, a defense function (phage growth limitation) originally identified in *Streptomyces* (269, 270). Seven configurations of *pglX* and *pglZ* “core” genes together with other annotations were distributed throughout prokaryotes. The original *Streptomyces* configuration was named BREX type 2. The most abundant, and now the best-studied, category was named BREX type 1. Homologs of the MTase PglX determine sequence specificity and provide the organizing principle for this review section (Table 2). BrxL is characteristic of type 1 systems.

**TABLE 2:** PglX (BrxX) components of BREX type 1 systems^*h*^

System^*g*^	REBASE^*f*^	References^*a*^	Sequence^*b*^	Uniprot^*c*^	NCBI^*d*^
Bce	M.Bce3081I	(18)	TAGGmAG	P0DUF3	EDZ57596.1
ECHS	M.EcoHSI	(271, 272)	GGTAmAG	P0DUF9	ABV04727.1
Lca	M.LcaZI	(273, 274)	ACRCmAG	P0DQP1	ADK19266.1
VchInd5	M.VchE4II	(275, 276)	RTAAmAYG	NA	AYV10810.1
EFER	M.EfeV	(202, 277)	GCTAmAT	B7L3T0	CAQ86937.1
StyLT7II^*e*^	M.StyLT7II	(278–280)	GATCmAG	A0A0D6HQC3	QTQ04188.1
Acin	M.BspHII	(281, 282)	GTAGmAT	A0A7H8SL17	QKY92361.1

^*a*^ References report *in vivo* characterization of BREX systems.

^*b*^ DNA sequence modified by the system.

^*c*^ Uniprot database record for the modifying protein.

^*d*^ NCBI Genpept record for the modifying protein.

^*e*^ Additionally, this table corrects misattribution of the REBASE name of the Salmonella MTase previously known as StySA; it is StyLT7II, not StyLTIII as in references 173–175. Present numbering adopts the convention of references 176, 177.

^*f*^ REBASE, REBASE enzyme name.

^*g*^ Short name used here.

^*h*^ Gene clusters containing the CDS are shown in Fig. 8; also note that Pgl/Brx nomenclature is not uniform in the literature.

**Fig 8:**
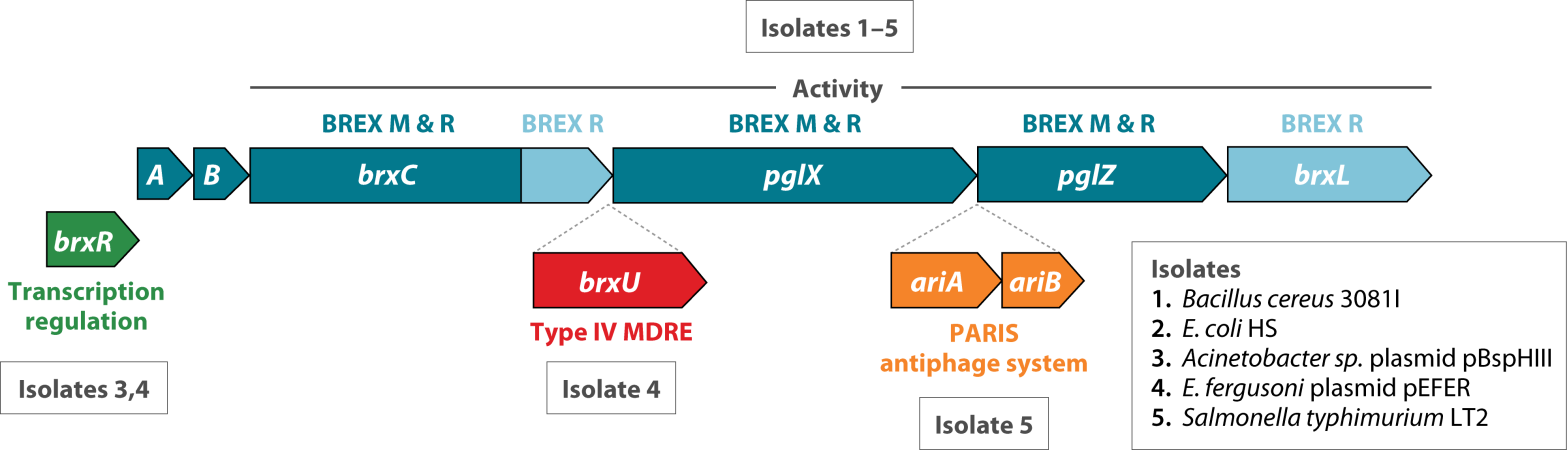
BREX gene clusters discussed. Block arrows represent genes that are present in BREX clusters, labeled with the gene name (A = *brxA*, B = *brxB*). Numbers identify strains listed at lower right. Each row corresponds to a set of genes found in one or more characterized BREX clusters; all five examples carry homologs in blue. Two plasmid-borne examples carry *brxR* (green). Extraneous genes not involved in BREX activity express a Type IV MDRE enzyme (red) or two genes specifying another antiphage activity (orange). Functional roles are unclear except for BrxR, shown to repress transcription; and PglX, with methyltransferase catalytic signatures. The five dark blue genes are required for both modification and restriction where tested; light blue genes (*brxL*) or segments (*brxC* C-terminus) are required only for restriction where tested.

Phage or plasmid methylation and restriction (exclusion) have been validated for seven type I systems (Table 2). Five type I BREX systems have been studied in some detail (Fig. 8). They share six genes, with additional adventitious genes in some cases. PglX/BrxX homologs carry signature catalytic motifs of DNA MTases. These are now annotated as MTases in REBASE (formerly listed as Type IIG). Where tested, PglX is required for both restriction and modification (as is observed for HsdM in Type I); this has been further validated by complementation in *trans* for *E. coli* HS BREX (271) and *Lactobacillus casei* Zhang BREX (273). Some mutations in PglX can prevent restriction while allowing modification (278), reminiscent of such mutations in EcoKI HsdM (182).

Some notable aspects of BREX biology are described below.

#### Establishment

BREX clusters have been readily transferred to naive hosts by natural transformation (18), Hfr transfer (283), and artificial transformation (202, 281). It may be significant that two conjugal plasmid-borne BREX systems (202, 281) are accompanied by a regulatory gene, *brxR*, that is also widely distributed among other mobile defense clusters. In the fifth case, heterologous plasmid constructs were easily introduced into naive cells (271).

#### Transcription

Transcription patterns among BREX systems are not uniform. In several cases, disruption of individual genes caused toxicity or accumulation of secondary changes. For *Bacillus cereus* (Bce), 5' rapid amplification of cDNA ends (RACE) identified operons *ABCX + ZL* (18). The *E. coli* HS (ECHS) cluster was studied with arabinose-regulated heterologous plasmids in K-12. A single contiguous fragment *ABCXZL* enabled the restriction of laboratory phages. Individual gene disruptions were tolerated only when divided into two operons on compatible plasmids, *ABC* and *XZL* (271). *Acinetobacter sp.* (Acin_BREX) was expressed only as *RABCXZL* either from native p*brxR* or heterologous p*tet*. Unusually, the *brxR* gene but not the BrxR protein was required for both HDR and HDM activity. Individual disruptions survived but were not always healthy (281). *Escherichia fergusonii* (EFER_BREX) has four promoters able to report LacZ activity in a promoter-probe vector: one at upstream *brxS* (not shown in Fig. 8), *brxR*, *brxA,* and *pglZ*, yielding expected transcripts *SR*, *R*, *ABCUX*, and *ZL* (202). For *Salmonella typhiumurium* (StyLTII_BREX), strong bidirectional terminators flank the chromosomal cluster, and an internal terminator (*brxCt*) is read-through at a low level by RNA-seq, enabling expression from *pglX*. Promoter activity measured by the detection of 5' triphosphate ends (CappableSeq) gave complex results. Principal starts mapped upstream of *brxA* and *pglZ*; additional internal starts and antisense transcripts were observed but not fully explored (279).

Curiously, it appears that when the StyLTII_BREX is transplanted into *E. coli* DH5alpha, the system does not require brxL for restriction and is sometimes more powerful without it (284). Since major changes in DNA metabolism accompany the *recA* deficiency of this *E. coli* host, there may be hints here regarding phage interactions with host DNA processing.

#### Methylation process

The progress of the modification event *in vivo* was studied with Bce_BREX. Infecting sensitive phage remaining unmethylated for at least a complete phage burst period (18). The ECHS_BREX cluster on a plasmid exhibited classic HDRM behavior, with phage λ passed through the restricting host resistant to restriction on reinfection, but sensitivity recovered following passage through the BREX- host (271). This confirms that the host-dependent modification pattern is crucial, excluding the possibility that rare phage survivors are mutants.

Methylation *in vivo* by M.StyLTII_BREX was proposed to require a complex of BrxC-N, PglX, and PglZ. This proposal is based on transcription patterns and *in vivo* modification properties of strains with mutations in (*brxC*, *pglZ*) or disruptions of the genes (*brxB*, *brxC*, and *pglZ*) (279). The BrxC-C terminal domain is dispensable for methylation.

Recent *in vivo* and structural work with the HDRM systems StyLTII_BREX (284) and ECHS_BREX (285) agree that the BREX MTases comprise two-domain, four-subdomain structures able to bind to SAM *in vitro* and shift DNA with unmethylated DNA sites in gel systems. Nevertheless, these assemblies cannot modify the site *in vitro* (285). Drobiazko et al. note that the original PglX of *Streptomyces coelicolor* A3(2), which does exhibit MTase activity *in vitro*, lacks the flanking N-terminal and C-terminal subdomains of the BREX MTases. These flanking domains distinguish BrxX from PglX and are implicated in the control of both modification and restriction.

Pull-down experiments showed the co-assembly of ECHS_BrxBCXZ *in vivo* (285). Domain swap and point mutation changes clearly identified the target site recognition domain (TRD) component of BrxX (284).

#### Restriction process

“Host-dependent restriction” describes the attack on entering DNA that depends on its history, read from modification marks made by the most recent host. This host-imposed pattern is monitored by a new host as the DNA enters the new cell.

As with Type I HDRM systems, the BREX MTase is the master of ceremonies, required for both methylation and restriction activity in collaboration with other components. Unlike classic Type I systems, the TRD is an integral part of the MTase protein (284). Variants in the M.StyLT7II_BREX PglX C-terminus unlink HDR and HDM phenotypes (278), as do some changes in the StyLT7II_BREX BrxC C-terminus (279).

Southern blot detection of phage-injected DNA showed that Bce_BREX aborts the growth of the infecting phage but does not lose the phage DNA over the course of the phage burst found with non-restricting control. This provides the best demonstration so far of non-cleavage (18).

The intracellular fate of infecting phage λ DNA was followed using fluorescent detection of protein:DNA foci inside *E. coli* with or without the ECHS_BREX system (271). Detection relies on focus formation by the tagged *E. coli* SeqA:Gfp protein. SeqA binds specifically to hemi-methylated Dam sites in DNA but not to fully modified or unmodified sites. When Dam-methylated DNA (5'GmATC on both strands) enters a Dam-deficient host, the first replication event yields a permanent hemi-methylation mark that can be tracked during cell growth, since the daughter cannot be modified (286). Fluorescent SeqA-Gfp foci normally form when Dam-methylated phage λ infects in the absence of BREX, divide into two, and then eventually dissipate as phage maturation proceeds. In contrast, no fluorescent foci were seen when Dam-modified BREX-sensitive phage λ infected a BREX + Dam-deficient host. Phage proliferation is thus aborted at a very early stage. Cell division was delayed and some filamentation was seen (271). Arrest so early suggests that BREX forms a first layer of defense. There is not enough time for defense expression to be launched after the invasion has begun.

#### Some component interactions

##### BrxR::DNA

BrxR is of particular interest due to the frequent association of WYL-domain proteins with diverse defense islands (277, 281, 287). In the case of Acin_BREX, the gene sequence but not the protein sequence is required for both HDR and HDM. The protein binds to a repeat element upstream of its own gene to dampen transcription.

BrxA is required for methylation in three cases tested, but not in the fourth tested by deletion. It is quite possible that the gene or protein is needed to allow the expression of the other genes or assist in the folding of the products. BrxA is a strong candidate for a DNA-binding role based on the crystal structure (288). A proposed relationship with NusB (18) is disfavored.

##### PglX:Ocr

The modifying complexes in EcoHS_BREX and the Type I HDRM EcoKI system may share conformation, as revealed by interaction with the phage T7 antirestriction protein Ocr. A study with both in the same cell found that both are thwarted by Ocr; T7 becomes newly sensitive to both HDRM when the gene (0.3) that specifies Ocr is deleted (272). Pull-down experiments demonstrated the binding of Ocr to BrxX(PglX) but not BrxZ(PglZ). Ocr is known to bind to M.EcoKI (289). Ocr also inhibits restriction by StyLT7II_BREX *in vivo* (290), further uniting the collection of BREX systems.

##### BrxL:DNA

The Acin_BREX system also provided a tantalizing glimpse of BrxL function compatible with action to disrupt the function of phage replication origins. This results from a combination of crystallography, cryo-EM, physical and enzymatic characterization, and mutational exploration (282). BrxL comprises three domains: an N-terminal domain with distant similarity to MCM proteins; an AAA+ ATPase domain like those in many multicomponent assemblies; and a C-terminal domain usually annotated as a Lon-like protease. This diagnostic C-terminal domain shows as much similarity to RadA, a DNA recombination enzyme, as it does to Lon. In fact, BrxL_C plays a key role in the ATP-dependent assembly of 12-mers forming a tunnel containing dsDNA. Assembly proceeds through an intermediate assembly of 7-mer rings, which are then converted to two tail-to-tail 6-mer rings upon binding to dsDNA. Curiously, only mutations that destroy the ATPase catalytic site yield a restriction defect with *in vivo* phage assays.

### Tantalizing questions for the future

What is the nature of the recognition site required to trigger “restriction”? Specifically, how do number and orientation of the modifiable target sequences affect restriction? Analogy with Type III enzymes suggests that a BREX system might detect site orientation by translocation.

What is the target of the putative phosphatase activity of PglZ? This is of particular interest due to the wide distribution of PglZ homologs first outlined in (18).

What is the target of BrxL activity and how is replication arrest mediated?

### Phosphorothioate (PT) modification as protection and target

#### What is it?

PT modification substitutes sulfur for non-bridging phosphate oxygen in the DNA backbone. Such substitutions may be familiar from laboratory use in chemically synthesized oligonucleotides to block nuclease action. However, phosphorothioate DNA can be synthesized *in vivo* using enzymes to do the chemistry. These are installed site-selectively and stereospecifically.

Genes for three core activities are clustered, with a fourth sometimes found elsewhere. The gene for cysteine desulfurase (*dndA*, *dptA*, and *sspA*) may occur with the others, or a conserved chromosomal gene *icsA* is found at a distance. Alternative sets of three core activities work together: a “phosphoadenosine-5′ phosphosulfate (PAPS) reductase” for sulfur transfer (*dndC/dptC/sspD*); one of two ATPase families (either an ABC/CXC ATPase *dndD/dptD* or a ring-forming ATPase *sspC* [291]); and one of two DNA-recognizing components (*dndE/dptE* or HTH family *sspB*). HDM activity may involve nicking of the backbone by DndE (292) or SspB (293) to prepare DNA for PT installation. A regulatory gene *dndB* is sometimes present.

**Fig 9:**
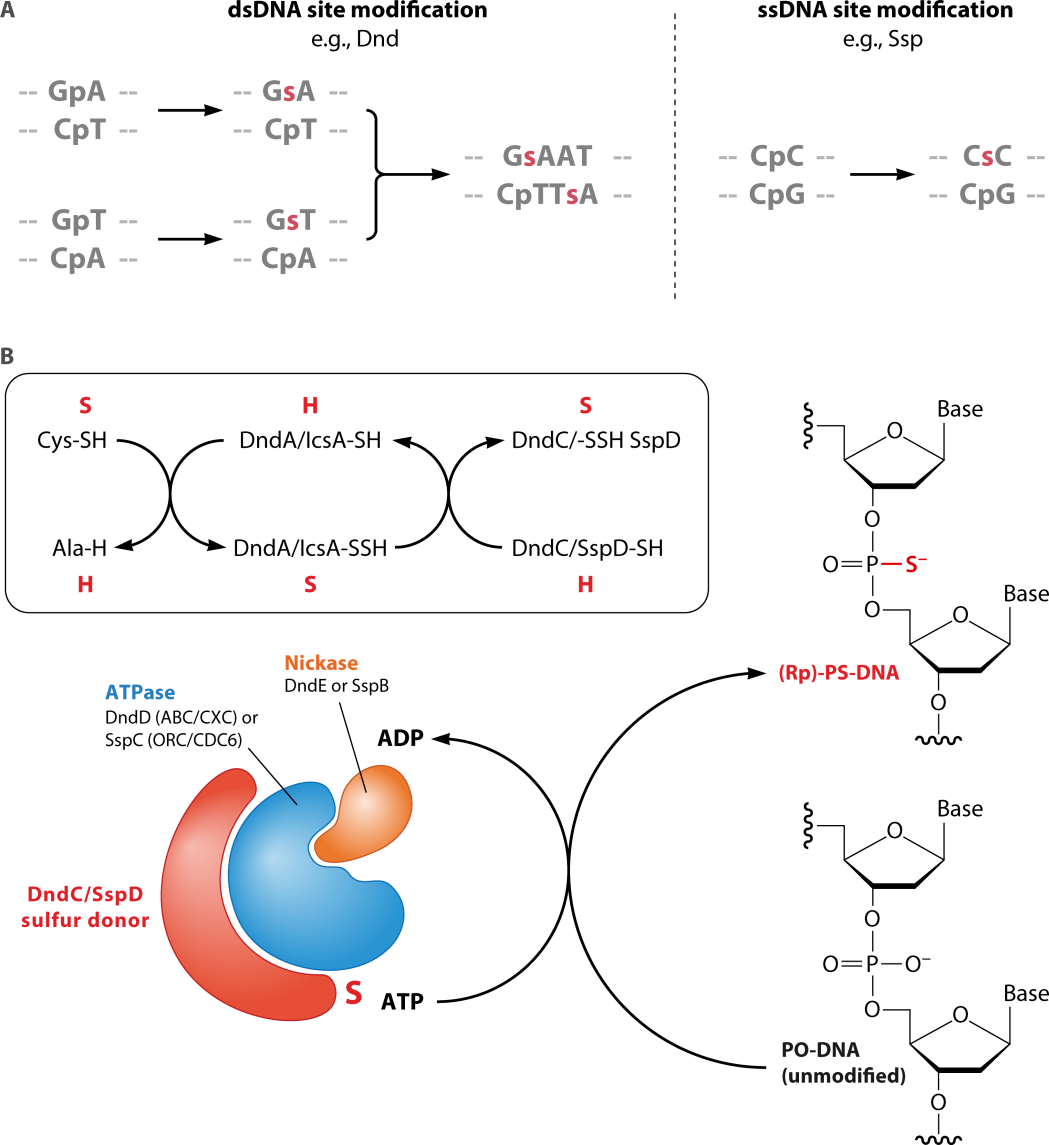
Biological phosphorothioate formation. (**A**) DNA sites modified by Dnd and Ssp systems. Dnd systems replace nonbridging oxygen in the phosphate moiety (p) with sulfur (s) preferentially in GA or GT dinucleotides. When both strands are modified, the consensus 5‘GAAC/5’GTTC becomes 5’GpsAApC/5'GpsTTpC. The resulting linkage is shown at the bottom right of panel B. Ssp systems modify only one strand (5' CpsC 3’), using a reader complex distinct from the double-strand capable Dnd systems. (**B**) Flow of sulfur (S) as a series of disulfide reduction steps begins with cysteine and finally transfers to DNA. Transfer is mediated by related or analogous activities at each step. The final sulfur donor (DndC or SspD) collaborates with alternative DNA nicking-ATPase components to provide energy and possibly substrate stabilization.

Sulfur from cysteine is donated to DndA or paralog IcsA, transferred to DndC/SspD, and then inserted by DndDE into dsDNA or by SspBC asymmetrically into an ssDNA site. DndB (not shown in Fig. 9) regulates transcription in *Streptomyces*. The first *in vitro* example (294) identified a three-protein DptCDE complex from *Salmonella enterica* serovar Cerro87 (corresponding to DndCDE in the *Streptomyces* system).

#### Gene distribution

Most thoroughly examined are *dnd(AB)CDE* as core components of biological PT installation at symmetric sites. In *sspABCD* (single-strand phosphorothioate) systems, functional substitutions enable installation at asymmetric sites (291, 295). The original phenotypic acronym (Dnd) refers to site-specific DNA degradation during gel electrophoresis in Tris-acetate-dependent on ferrous ions in the buffer (296).

Site-selective DNA backbone phosphorothioate modification (PT modification) plays a variety of roles in prokaryotes, like but distinct from that of base methylation (14). “Orphan” or “solo” modification capacity (HDM) occurs widely (291, 297), as also found with methylation-directed systems (see “Type IV systems: action when sites are modified”). A physiologic role may be the regulation of oxidation state. Solo modifiers are most frequent in Cyanobacteria (291), consistent with the idea that PT addition to DNA originated as an antioxidant capacity to cope with the Great Oxygenation Event (298), with subsequent repurposing. A second effect of PT modification can be gene expression changes (299, 300) as also found with changes in base methylation (11). A third role is the regulation of DNA entry from nonrelatives, HDRM. As with conventional HDRM, these systems are distributed sporadically throughout the prokaryotic tree and prevent entry of unprotected molecules (300, 301). As with conventional methylation marks, Type IV are also found—MDRE that target PT-containing sites (210, 267), facilitating identification of modification patterns (268)

#### PT distribution in the genome

A key difference between PT modification systems and MTases is that PT modification is found in only a fraction of susceptible sites at any time, whereas base methylation of specific restriction targets is typically >95%. *S. enterica* serovar Cerro 87 and *E. coli* B7A have highly similar gene clusters (302). In both genomes, GAAC/GTTC are modified on one or both strands, generating GpsAAC/GpsTTC, GpsAAC/GTTC, or GAAC/GpsTTC. However, only a fraction of potential sites are modified (303, 304). The modification level is not affected by overexpression of the HDM gene cluster (305), although its absence causes severe disruption of cell physiology when HDR is present (306). Interestingly, base methylation and backbone PT modification may affect each other: genetic manipulation of the Dam methyltransferase and the Dnd components revealed a redistribution of PT modification among sites, but maintenance of the same overall level of PT incorporation, suggesting a measure of homeostasis (299).

#### HDM: Modification enzymes

A gene for a universally distributed sulfur-management protein, cysteine desulfurase, may be tightly linked to (*dndA*, *dptA*, and *sspA*) or distant from (*iscS*) the cluster of DNA modification components. The regulatory gene *dndB* is sometimes present (307). The core HDM gene set of three activities mediates installation of the sulfur: a PAPS Reductase for sulfur transfer (*dndC/dptC/sspD*), an ATPase (either an ABC/CXC ATPase *dndD/dptD* or a ring-forming ATPase *sspC* [291, 308]), and a DNA-recognizing component (*dndE/dptE* or HTH family *sspB*) are found together (300, 309). HDM activity may involve nicking of the backbone by SspB (293) or DndE (292) to prepare DNA for PT installation mediated by DndCD (PAPS reductase, DndC; ATPase, DndD) (292); a radical-SAM mechanism has also been proposed for the Dnd system (310). Enzyme names attached to steps in the process vary between the dsDNA and ssDNA systems: SspC = DndD (ATPase, but of a distinct family, ORC/Cdc6 rather than ABC), SspD = DndC (PAPS Reductase) (293).

Recent bioinformatic refinement of ATPase domain assignment (308) has contributed to the expansion of the universe of PT-modification gene clusters (291). Sister clades carry relatives of ABC_ATPases DndD or CXC; in this view, each ATPase is paired with a coevolving site-determining protein (DndE), broadening the recovery of HDM clusters. This refinement still agrees with earlier literature (292, 305, 311) that DndE homologs are involved in interaction with DNA.

#### HDR: Restriction

Three distinct functional HDR restriction branches have been proposed: *dndFGH* (267, 312) (and homologs *sspFGH* [295]), *sspE* (313), and *pbeABCD* (295). In some cases, expression of HDR components DndFGH (306) is toxic in the absence of the HDM component. The SspE HDR component, a DUF262-DUF1524 homolog (313), appears to arrest growth without its HDM (293). HDM and HDR clusters can sometimes be exchanged; HDM may collaborate with two HDR components with additive activity (309). However, a deeper and more comprehensive bioinformatic analysis has questioned the idea of concerted evolution of HDR with HDM distribution and specificity (291).

In some cases, a single organism carries both kinds of clusters, as in the example of *Natrinema thermotolerans* A29. This organism has a *dndAEDC* HDM cluster, with an adjacent HDR cluster the topic of investigation. Rakesh et al. reannotated this to impute to PbeCD a DNA recognition function parallel to DndDE (in CXC ATPase branch). In this reannotation, the PbeAB components are assigned nuclease and SF-helicase functions likened to Type I and Type III enzymes. Independent evolution of the HDR and HDM components of this system and its relatives is agreed upon the two groups (291, 295). The nature of the HDR event remains obscure.

#### Phage and plasmid restriction and intracellular events

All the pieces need to be present in the right amount in several cases: overexpression of *dndC* and *dndD* (installation collaborators) of *Streptomyces lividans* strain 66 in *E. coli* appeared to be toxic but not lethal to the host (312).

In favor of the “host-protective marking” role for PT, the *S. enterica* Dpt system (267) reduces plasmid transformation from unmodified sources. In addition, deletion of *Salmonella* protective modification cluster *dptBCDE* caused severe disruption of cell physiology, including SOS-induction of DNA repair response when the HDR component *dptFGH* is still present (306). This behavior is expected when DNA cleavage is the primary toxic event.

Suitably PT-modified plasmids and phage are protected from restriction *in vivo* by DndFGH in *Salmonella*, measured by colony or plaque formation. The magnitude of *in vivo* restriction was obtained by comparing chromosomally encoded HDM-HDR intact versus deletion of both components. This is about 10-fold for plasmid transformation in both *E. coli* and *Salmonella* and about 104-fold for phage in *Salmonella* (302). Curiously, none of the three individual HDR deletions (*dndF*, *dndG*, or *dndH*) completely eliminated restriction, suggesting independent rather than collective action of the HDR components.

Intriguingly, the adenine-modifying type I BREX systems share with type IV BREX PT systems (*brxPCZL*) the action of a BrxC homolog in coordinating the action of the modification activity (314). The results raise interesting questions about communication between the modification activity and a suitable R activity. We may also query whether the tail of BrxC is involved in that coordination.

## HDRM AND GENOME ORGANIZATION AND TRANSFER

### Temporal studies of HDRM establishment in new host cells

Given the potentially lethal activities of restriction-modification systems (115, 306, 315, 316), one would expect them to be under very tight regulatory control: make enough HDR to repel invaders but not so much that HDM cannot keep up. Furthermore, the appropriate ratio of toxic and protective activities may not be constant when the cell faces major environmental shifts and physiological stresses (229, 317, 318). Even where the HDRM functions as a selfish/addiction toxin-antitoxin system (76, 117, 319), the regulation is optimized to minimize toxicity in all situations except for the case of gene loss. In addition to the general risks of carrying HDRMs, there is a particularly high risk associated with introduction into a new cell.

In this section, we review studies illuminating strategies and mechanisms for maximizing HDRM establishment and maintenance in host cells. This section focuses on regulation intrinsic to the HDRM itself, but the reader should be aware that a variety of external elements can also modulate HDRM activity. These range from a cell-controlled proteolytic process in response to UV damage called restriction alleviation (RA) (146, 147, 188; see “Type I HDRM systems: asymmetric sites and two-strand modification”) to phage- or plasmid-coded proteins that inhibit restriction activity (e.g., 32, 320; see “Anti-RM activities and host countermeasures”). Another form of REase activity control we do not consider in detail here theoretically provides significant, if limited, protection from cleavage of a host cell’s own DNA—a requirement for recognition of two sites at the same time, as found for the Type IIE REases (1, 121, 122), Type III enzymes (128, 131), and at least one Type IV enzyme (123; see “Organization and function of protein domains in classical HDRM Types I–IV”). Finally, there are several cases in which the MTase of a phase-variable Type I or III RMS has been adapted to play regulatory roles outside of the HDRM itself. These phase varions are fairly widespread and rely on slipped-strand mispairing in homopolymeric sequences to eliminate or restore expression of the HDRM (20, 21, 321; see “Effect of HDRM inventory on clade structure”).

Despite the substantial findings summarized below, much remains to be learned. This is due in part to the huge and growing number of unexplored HDRMs (2) that may have novel regulatory mechanisms. This is even true for canonical HDRMs, let alone the rapidly growing set of other phage defense mechanisms (14, 27, 309, 322–324). Furthermore, the appropriate regulation for a gene depends on its roles in the cell (285, 325–329), and it is not clear how high the expression of HDRM genes must be for phage defense (113, 330). Furthermore, phage defense may not be the only relevant activity (15, 20, 318, 331, 332).

#### Events

HDRM gene entry into new cells is a potentially lethal event, as the recipient cell has a completely unprotected genome. By some means, the RM system must ensure that host DNA protection (usually DNA methylation) precedes the production of the destructive enzyme (REase). This is particularly true of HDRMs carried by mobile genetic elements such as plasmids, but as described in “Effect of HDRM inventory on clade structure,” diversification of the HDRM components may itself be positively selected. Additional evidence for extensive horizontal gene transfer is found in many sequence analyses (4, 5, 333–337).

In this section, we review studies in which HDRM genes are transferred into new cells. We look forward to further such studies, making use of fluorescent protein fusions (338), and newer techniques such as have been developed to study kinetics of phage gene expression (339).

Type I systems: When the Type IA system EcoKI is conjugationally transferred into a new host cell, DNA methylation begins very rapidly, whereas restriction appears only after ~15 generations and is maximal only after yet another 15 generations (340, 341). The mechanism behind this extensive delay has not yet been clearly determined, but it seems likely that contributions may be made by mediated by ClpXP proteolysis (190, 342), possibly phosphorylation of the REase subunit (343), and the need for high levels of free MTase subunits for REase-active complexes to form (344, 345).

As discussed in “Type I HDRM systems: asymmetric sites and two-strand modification,” control mechanisms differ among distinct Type I subtypes. The Type IC HDRM EcoR124I can be introduced into a new host even if that host already contains the REase subunit gene *hsdR*, and the timing of the appearance of restriction is not affected by *hsdR* pre-expression (191). This is different from the case of Type IB EcoAI, for which pre-expression of *hsdR* does kill the recipient when the remaining genes are introduced (180). This phenotype is countered by a roughly mapped host gene designated *hsdC* (340), which is shown to be the protease component ClpX (147, 188).

Type II systems: As noted earlier, the need for delaying REase activity following HDRM transfer is particularly acute for those Type II HDRMs where the REase is a separate, independently active protein. This has been studied both in batch cultures and at the single-cell level. In batch culture, the main technical problem is the coordinated introduction of the new HDRM genes at time zero. This can be done by conjugation, although gene transfer will not be instantaneous, or by transformation or electroporation, although at the cost of subjecting the recipient cells to major physiological stresses in making them competent. Nevertheless, such studies have provided useful insights. In another case, the HDRM genes were cloned into bacteriophage M13, which does not kill the infected cell and minimally affects its gene expression pattern (346, 347). In that study, the delay between PvuII MTase and REase transcription was just under 10 min (348). Modeling and experiment later showed that a key variable in that regulatory system was the gene copy number–which makes sense for HDRMs on plasmids that enter in a single copy and then amplify–and the system showed hysteretic behavior, such that once the REase was expressed its gene tended to stay transcriptionally active (349).

The Esp1396I REase and MTase genes were fused in catalytically active form to fluorescent reporter proteins, put onto a high-copy plasmid, and introduced into new cells via electroporation (350). They found a (roughly) 1 h REase delay–specifically, the MTase rose after 10 min, rising to ~40-fold over the background after 2 h, before dropping 4-fold at about the same time the REase began a slow, steady rise, converging with MTase levels after ~3 h.

To our knowledge, the temporal behavior of just one HDRM has been studied using both batch and single-cell approaches. For Csp231I, in which the activating C protein controls only a fraction of REase transcription, the batch culture showed a decreased delay between MTase and REase (from 30 min to 15 min) when the C gene was deleted (351). In single-cell analyses, the wild-type (C+) system showed a 10 min average REase delay relative to MTase, with 83% of cells expressing REase later than MTase. In contrast, without C, REase expression occurred 15 min before MTase, with 85% of cells showing REase before MTase expression (351). The Csp231I study, which used the phage M13 delivery system, was made possible by the REase gene having a noncatalytic mutation, with both the REase and the MTase fused to fluorescent reporter proteins. It is interesting that in both reporter studies, the Esp1396I and Csp231I studies independently showed the MTase spent more time near the nucleoid than did the more widely distributed REase (350, 351), and, in this respect, the Csp231I C protein behaved like the MTases. Regarding localization, it is interesting that RNA:DNA hybrids play a role in nucleoid localization of the orphan MTase DnmA (352).

Type III systems: Curiously, one of the first systems to be described appears to be an exception to the idea that RM systems are adapted to travel. Genome surveys find that M.SenTFI (aka StyLT7I) is very stable in *Salmonella*, unlike other HDRM MTases (353, 354). The Type III system of which it is a part was one of the first HDRMs identified. It was extensively characterized using phage (355, 356) and was cloned using phage restriction assays in two steps (357–359). The system is resistant to transfer, both by conjugation (356) and by molecular cloning (357). The modified site fits the Type III category (358). Conjugation of the system into un-premodified HB101 led to extensive DNA degradation (357). HB101 is a Mrr-deficient host (360); hence, recalcitrance to cloning was NOT due to the action of the K-12 *mrr* allele being elicited by the LT2 *mod* function (361).

Other Type III HDRM appear to have evolved to facilitate the establishment of new hosts. EcoP15I can move into new host cells–*mod* and *res* genes are transcribed at the same time after entering a new cell, but the translation of *res* is delayed until after protective modification is present. Res activity may require the presence of Mod (HDM) for the toxic Res (HDR) protein to be active (103).

### Effect of HDRM inventory on clade structure

It has long been understood that HDRM systems undergo frequent horizontal gene transfer (HGT). Evidence supporting this includes differences in base composition between HDRM system genes and the host core genome (335), linkage with mobile elements and mobility-promoting genes (362–364), and significant differences in HDRM inventory between closely related strains (365, 366). HGT thus results in a “patchy distribution” of HDRM systems across phylogenetic trees (367). Put another way, most HDRM system genes belong to the accessory—as opposed to the core—genome of any taxonomic group in which they are found.

Two advantages result from this HGT-driven diversity. One results from the protection afforded by the restriction function: no one bacteriophage can sweep through a population that carries diverse subsets of “defense” identities conferred by distinct modification patterns. The second relies on the modification function itself: DNA methylation changes the nature of the major groove, where transcription regulators act. Altered transcription resulting from the gain or loss of a DNA MTase gene can potentially alter the expression levels of many genes simultaneously (21, 321). Epigenetic diversity within a population resulting from a fluid inventory of DNA MTase genes may produce advantageous phenotypes adaptive in changing environmental conditions.

HDRM systems are not simply the passive objects of HGT. Once established in a host, they themselves have a profound effect on the HGT process, directing and limiting it. Epigenetic patterns, comprising base modifications or phosphorothioate additions to the backbone, are laid down by the modification function; these define what the cell considers to be “self” DNA (368). The restriction function then filters and shapes (typically by nucleolytic digestion) any “non-self” DNA subsequently acquired by that host. Thus, HDRM systems are both “immigrants” that arrive from outside the cell and “immigration control agents” that determine who is allowed entry after them.

HDRM systems act on all three major modes of HGT: transduction, transformation, and conjugation (369–371); a fourth mode (vesiduction [7]) has not yet been tested in this regard. Since restriction endonucleases typically act only on double-stranded DNA, the degree of restriction is greatest for phage-mediated transfer, where double-stranded DNA enters. Such DNA may comprise the phage genome or transduced (accidentally packaged) chromosomal DNA. Conjugation and natural competence invade with the entry of a single-stranded molecule (370), resulting in a smaller effect (369, 372–375). Nevertheless, the entering molecule must become double-stranded for permanent establishment, and restriction still applies pressure in these cases. Indeed, some MTases have evolved to target single strands, leading to the protection of conjugal transfer (29), recombination intermediates (376), and transformation intermediates (377). There are, of course, bacteriophages with RNA or ssDNA genomes (378).

What a cell considers to be “self” is any DNA, regardless of evolutionary origin, with epigenetic marks compatible with its own set of HDRM systems, although the level of sequence relatedness can also strongly affect the fate of the DNA (379). The systems identifying DNA as self for a given cell create siloed sets of trading partners that are genetically isolated from incompatible donors and recipients outside the bloc. It was speculated in 2003 that this HDRM-driven genetic isolation may be a significant force in microbial evolution, perhaps leading to speciation and the creation of smaller-order taxonomic structures (380). Such smaller-order structures have since been observed in multiple species. In *Neisseria meningitidis*, strains are organized into phylogenetic clades (PCs), each with a unique repertoire of HDRM systems of all types (381). A similar phenomenon was observed in *Burkholderia pseudomallei*, where whole genome sequencing revealed genomic clades, each with distinct methylation profiles; members of different lineages were co-isolated from the same metagenomic samples, indicating that HDRM systems limited genetic exchange between sympatric strains of the same species (382). In *Enterococcus faecium* a subclade-specific Type I HDRM system (Efa502I) was identified that acts as a barrier to HGT and may contribute to subspeciation (383). A Type I HDRM system (originally designated Sau1) also limits genetic exchange between lineages of *Staphylococcus aureus*, with different specificity subunits defining, or at least correlated with, these lineages (384).

These examples show how, in multiple species, HDRM systems can create barriers to genetic flux between lineages with incompatible epigenetic marks and enable preferential routes of genetic exchange within and between compatible lineages. More generally, Oliveira and coworkers have shown that, in a collection of 79 bacterial species, both HGT and homologous recombination events increased with the number of HDRM systems (385); however, this effect could not be decoupled from genome size, since larger genomes tend to encode more HDRM systems. More clearly, they showed that lineages encoding compatible HDRM systems coexchanged more genetic information than average (385).

Whether these HDRM-mediated lineages lead to bacterial speciation depends on how long they can be maintained. Although the ages of *N. meningitidis* phylogenetic clades are estimated to be on the order of decades (381), there is little other evidence at present to say how long such lineages persist in general. Lineage establishment could well be a precursor to speciation if it lasts long enough for genetic isolation to be reinforced by other factors such as physical isolation. There is evidence of cryptic speciation in the cyanobacterium *Lapsinema thermale* due to barriers to gene flow, but it has yet to be shown that this barrier is HDRM system-related (386). On the other hand, the general pattern of HDRM evolution favors diversification across populations, meaning these systems are acquired and replaced rapidly (367, 380). The gain or loss of new methylation patterns can disrupt incipient lineage arrangements, potentially breaking them down before the reinforcement of genetic isolation could happen. For example, *Neisseria gonorrhoeae* cells are killed, when they accept DNA with foreign methylation signatures derived from other *Neisseria* species, during the process of genetic transformation (387). Similarly, HDRM-dependent, differences in DNA methylation status affect DNA acquisition by *Acinetobacter baumanni* cells. Overall, intra- and inter-species DNA exchange in naturally competent bacteria appears to be strongly dependent on methylation patterns, in addition to the activity of competence regulators (388). Furthermore, once genetic isolation is enforced by other factors, the HDRM systems that established the incipient lineages could be lost, along with the evidence for their role in the process. More work is necessary to identify convincing cases.

### Defense islands: laboratories of evolution and exchange

Early in the sequencing era, researchers expected a fair amount of chromosomal stability in bacterial genomes. *E. coli* and *S. enterica* sv Typhimurium (the best-known model organisms) could exchange genes by conjugation, yielding a similar gene order at many loci. This expectation was dispelled slowly, then suddenly. Early work compared samples from three *Salmonella* isolates with "the" *E. coli* MG1655 sequence, noting large clusters of missing genes (280). Then, a comparison between the first complete *E. coli* sequence (MG1655) with the second (O157:H7), yielded almost as much (25% of O157:H7 genes) presence/absence variation between strains as between the different genera (389). These gene clusters were dubbed “genome islands,” contributing to the species “pangenome” (all genes found in any isolate) but not the “core genome” (genes common to all natural isolates in a taxonomic grouping).

**Fig 10:**
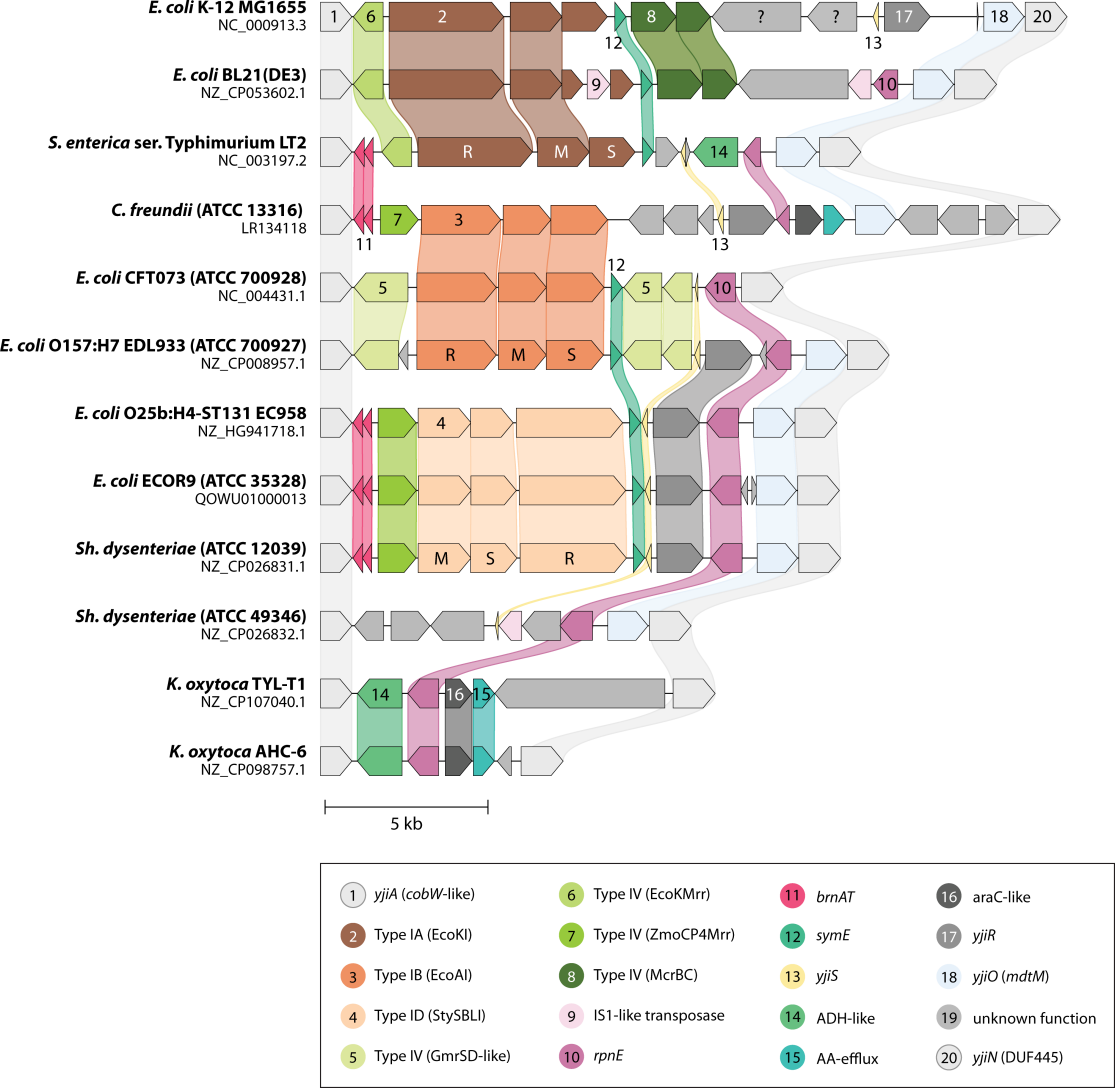
The enteric immigration control region (ICR) exemplifies a defense island. Gene diagrams of the ICR from select enterobacterial strains. The gene content between the conserved boundary genes *yjiA* and *yjiN* is highly variable across the *Enterobacteriaceae*. Most loci include genome defense functions, including three families of Type I restriction endonucleases (*hsdRMS*) and Type IV modification-dependent restriction endonucleases (e.g., *mrr*, *mcrBC*, and *gmrSD* homologs). Apparent non-genome defense functions encoded in the ICR such as hydrolytic enzymes (penicillinase), metabolite reductases (aldehyde dehydrogenase), and small molecule transporters (*mtdM*) encoded in this locus may have additional fitness effects.

**Fig 11:**
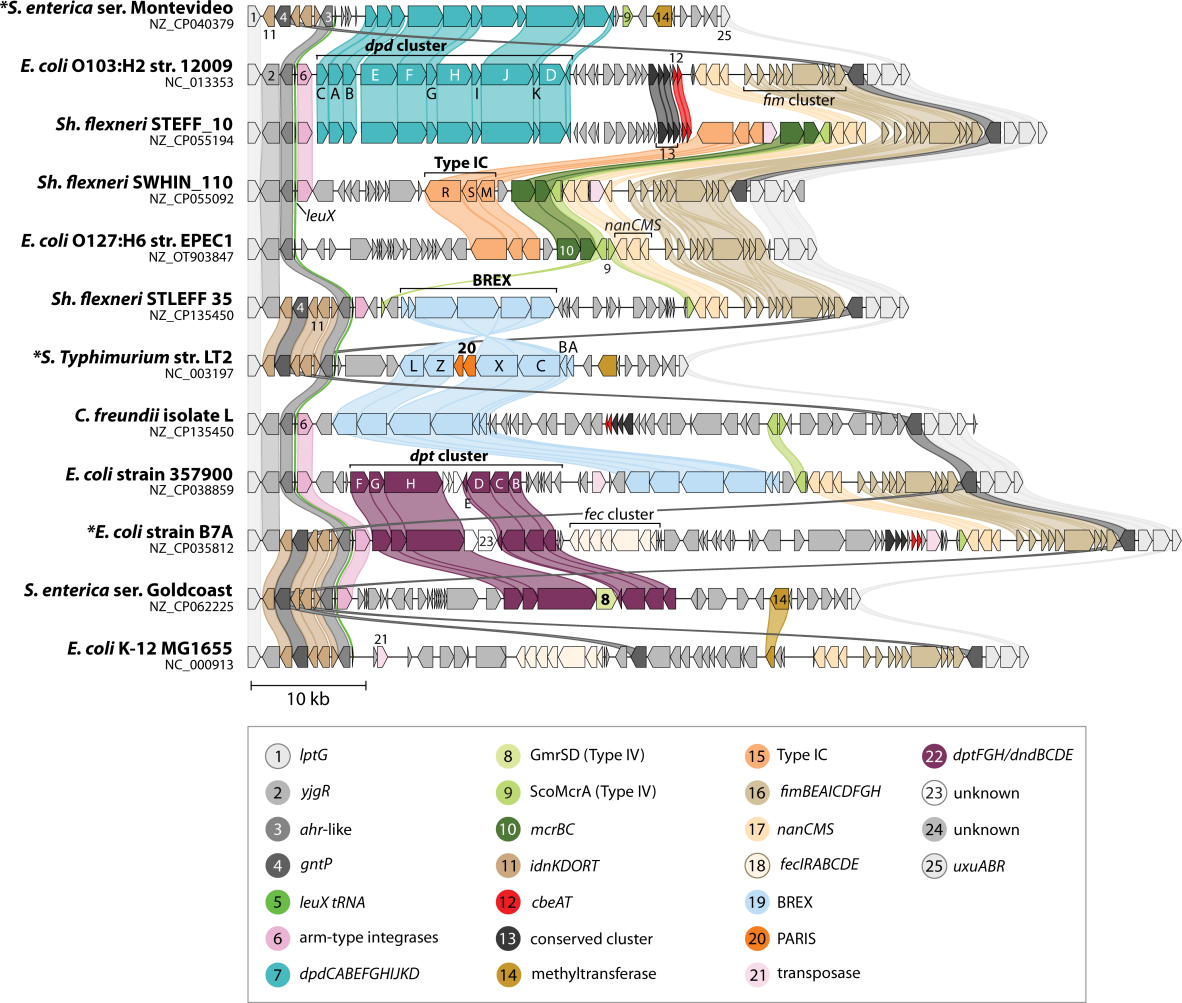
Hotspot (HS)37 extended to *Enterobacteriacaeae*. Gene diagrams of hotspot #37 from select enterobacterial strains. This hotspot is anchored by the *leuX* tRNA, an attachment site for a site-specific integrase. The spectrum of defense and metabolic activities found here is like the ICR, but family memberships are distinct.

The tendency of HDRM systems to cluster genetically was observed early (6) as was the action of HDRM in shaping the outcome of homologous recombination in *E. coli* (390, 391). Among other things, Milkman’s work identified two chromosomal regions of high sequence variation in wild isolates (Bastions of Polymorphism) and proposed that variation itself was a selected property. One variable region contained HDRM genes. This region included the immigration control region (ICR) and very possibly the nearby “hotspot #37” (Fig. 10 and Fig. 11; also see below). The second variable island is required for the synthesis of the highly diversified cell surface O-antigens (391). These outer membrane sugar chains are recognized by mammalian immune cells, protozoa, and many phages (392). For both HDRM and cell envelope elaboration, biotic competition between organisms appears to reset the successful genotype frequently.

Overall, defense systems act combinatorially, under selection for novelty. A broad set of defense systems with elements variable within populations, and exchangeable between related groupings, yields the best likelihood of survival in the case of predation or parasitism (153, 393, 394). Given that no one species can encode all defense systems potentially available to it, the diversity of defense functions found within any bacterial pangenome likely is the result of two forces: “killing the winner” (395, 396) in which efficient resource use attracts parasitic attack and “Red Queen” (running as fast as you can just to stay in the same place) (397–399). Which dominates can vary with niche characteristics (400). The evolution of defense strategies evidently must be driven by biotic interactions rather than physical adaptation. These are examples of negative frequency-dependent selection in which a rare genotype is favored (401).

The ICR at ~98 min on the *E. coli* K-12 genome (coordinate ~4.6 Mb) (Fig. 10) was the first defense island investigated in some detail (23). It was defined by genetic identification of an “empty site” from *E. coli* C. *E. coli* C was the “restrictionless” strain that defined the host-dependent restriction phenomena of *E. coli* K-12 and B (136). Comparison of the “empty” C sequence with an early set of complete genomes identified conserved anchor genes bordering the variable locus.

An updated illustration of the ICR in Fig. 10 extends the taxonomic scope to *Enterobacteriaceae*. In the *E. coli* str. K-12 substr. MG1655 genome (Genbank accession U00096.3), this region spans nucleotides 4,565,966–4,588,865 or between 98.4 and 98.8 min. There is a high degree of variable content in enteric bacteria between anchors *yjiA* and *yjiN*. Out of 9,040 closed/compete genome assemblies belonging to the *Enterobacteriaceae* (taxid = 543; retrieved in January 2024) 8,424 records (93%) contained a genomic segment recognizably bounded by these conserved anchors. The role of *yjiN* is not yet clear, but *yjiA* is a GTP hydrolase (402). Loci chosen for display in Fig. 10 have genetic evidence for functionality where possible, filtered for closed chromosomes, and to illustrate the presence of independent family structures: Types IA, B, and D HDRM system families (see “Type I HDRM systems: asymmetric sites and two-strand modification”) are accompanied by Type IV MDRE genes (see “Type IV systems: action when sites are modified”), and appear to have evolved in separate lineages. These three distinct Type I (HsdRMS) families are accompanied by three distinct Type IV families as well as three toxin-antitoxin systems (*brnAT*, *symE symR,* and *rpn* [403–405]). Not found in the 9,040 loci in the set analyzed were Type IC systems nor were the relatives of BREX, PT, or ADG-protected systems (Table 3).

**TABLE 3:** The ICR and HS37 host distinct major HDRM and genome defense systems

Defense island	Defense system	Frequency
ICR (Hotspot 36)	Type I (all types)	36%
ICR (Hotspot 36)	Type IA	14%
ICR (Hotspot 36)	Type IB	11%
ICR (Hotpsot 36)	Type ID	12%
ICR (Hotspot 36)	Mrr	22%
ICR (Hotspot 36)	McrBC	6%
ICR (Hotspot 36)	GmrSD	12%
Hotspot 37	Type I (all types)	17%
Hotspot 37	Type IA	3%
Hotspot 37	Type IB	7%
Hotspot 37	Type IC	4%
Hotspot 37	Type ID	3%
Hotspot 37	Dpd (7-deaza-dG)	2%
Hotspot 37	BREX	9%
Hotspot 37	Dnd (PT)	2%
Hotspot 37	DISARM	12%

Another defense island is quite near along the genome to the ICR but very distinct in content. The *E. coli* “hotspot #37” (406) (Fig. 11) is anchored by the tRNA gene *leuX*, one of several tRNA loci targeted by mobile element integrases (407); the *leuX* region has long been known for hypervariable content (408). It is the most highly occupied locus among 41 identified *E. coli* defense islands; 97% of isolates contain defense genes here. As with the ICR (“hotspot #36”), gene clusters specifying functionally collaborating systems appear here, with replacement (Fig. 11). In contrast to the ICR, however, the mobilizing activity is present at one anchor, the tRNA-recognizing arm-type site-specific integrase near the left end.

Within HS37 are examples of HDRM systems not found at the ICR: the Type IC family, BREX defense, and PT-related defense. The ADG-protected RM cluster (*dpd*) carries the well-characterized modifying component *dpdCAB* (409), together with a presumptive restriction cluster. The eight associated genes were found to reduce transformation efficiency when transplanted into a naive context in *Bacteroides* (12).

The two islands display distinct sets of defense genes (Table 3). Out of 9,040 complete enterobacterial genomes (taxid = 543) downloaded from NCBI during January 2024, 8,424 (93%) contained an ICR region bounded by *yjiA* and *yjiN*. Similarly, 6,142 (68%) genomes yielded a "Hotspot 37" bounded by *lptG* and *uxuR*. Major genome-defense functions encoded therein and their frequencies are listed here.

## ANTI-RM ACTIVITIES AND HOST COUNTERMEASURES

Bacteriophages, plasmids, and infectious conjugal elements are collectively known as mobile genetic elements (MGEs) (385, 395). These are targets of HDRM systems, which themselves have diversified via horizontal exchange as described in “Type I HDRM systems: asymmetric sites and two-strand modification and HDRM and genome organization and transfer.” Successful entry of an MGE may bring advantages to the host (metabolic capacity including drug resistance or adhesive properties [410]). On the other hand, death or intracellular conflict may also result (411, 412). Thus, MGEs employ a wide variety of strategies to circumvent the activity of HDRM systems, from avoiding restriction sites in their genomes, to shrouding their DNA with chemical modifications, to encoding proteins that directly inhibit restriction endonucleases. In turn, this plethora of evasion mechanisms creates vulnerabilities that are exploited by cellular defense systems. Many questions remain unanswered and are highlighted below for the next 10 years and beyond.

### Passive antirestriction

**Fig 12:**
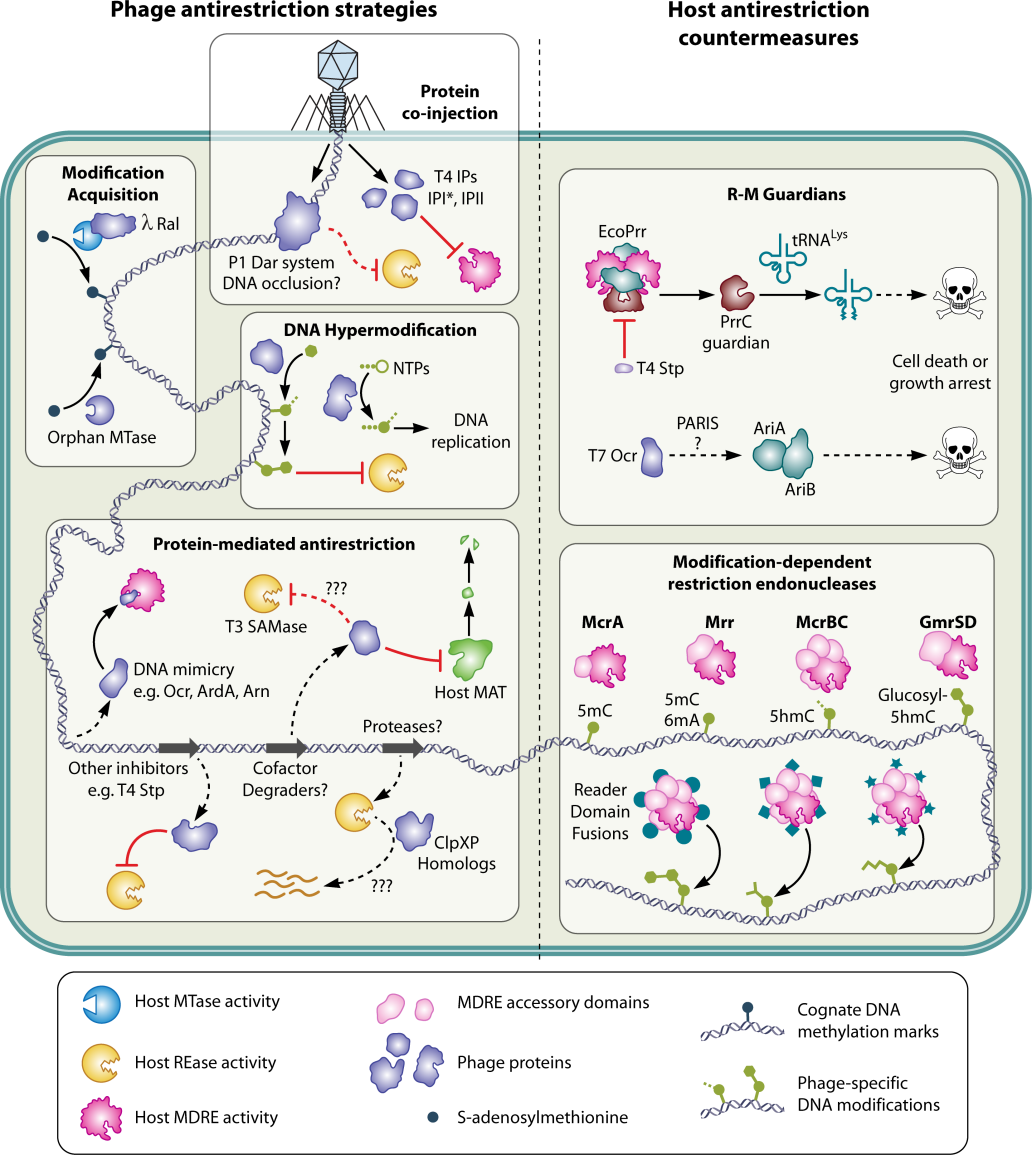
Phage antirestriction activities and host countermeasures**.** Bacteriophages use a broad range of strategies to interfere with host restriction-modification systems (shown in boxes, left). On entry, these include phage DNA co-injection with protein inhibitors of RM, early expression of genes encoding proteins that stimulate the acquisition of host modification patterns, and prior chemical modification of phage DNA to shroud it from RM recognition. During development, a variety of encoded proteins block restriction through diverse means. In response (on the right), bacteria may encode additional immune systems that directly target antirestriction countermeasures. RM guardian systems such as PrrC and PARIS detect RM inhibition and activate abortive infection responses that drive cell death or growth arrest. Type IV MDREs (modification-dependent restriction endonucleases) directly target phage DNA modifications and hypermodifications.

Types I–III, BREX, and PT-protected HDRM systems discriminate self from non-self at the DNA level epigenetically (Fig. 12). As illustrated, the host genome is methylated, and unmethylated DNA is destroyed by restriction endonuclease cleavage. Although this strategy represents the most widespread known mechanism for anti-MGE defense (8), restriction-modification systems have a variety of weaknesses, which are exploited by MGEs for HDRM evasion.

#### Removal of recognition sites from genomes

One strategy for HDRM evasion is to purge genomes of HDRM motifs. Bioinformatic analysis of phage genomes and known modifiable sites (413) found that DNA phages with a virulent lifestyle show depletion of Type II modification sites (414), as the probability of bacteriophage escape increases with each mutated restriction site, as shown experimentally using phage λ mutants and EcoRI the Type II system (415). The same sites (short 4-6bp palindromes) are also underrepresented in DNA of small and conjugative plasmids (416, 417). However, the inventory of modifiable sites may over-represent the inventory of effective restriction sites. Where HDR mechanisms depend on the recognition of multiple sites, many modifiable sites may be present yet not constitute a restriction site (120, 290) (Fig. 5).

#### Occlusion of restriction sites

Instead of purging HDRM sites from the genome, chemical modifications can block site recognition and/or action by HDR enzymes. This strategy is frequently employed by virulent phages for evasion of action by HDRM systems (33, 418–420). Phages are known to employ modifications to all 4 DNA bases. Most frequently, the pool of nucleoside triphosphates is purged of one type of base and a derivative base with a reactive chemical handle is incorporated during replication. The chemical handle is then further derivatized post-synthetically with a range of metabolites: sugars, amino acids, or organic compounds like putrescine (33). The earliest-characterized examples were *E. coli* T-even phages that convert CTP to 5-hmCTP (421). This derivatized nucleotide is then incorporated into phage DNA and may be further decorated with sugars resulting in α− or β-glucosyl-methylcytidine (T4) or gentiobiosyl-methylcytidine (T2 and T6) in polymerized phage DNA (422). A related strategy acts on TTP to incorporate modifiable 5-hmU into phage (36).

Most recently, a post-replicative mechanism has been described: 7-deazaguanine derivatives are incorporated into the DNA via a transglycosylase homolog (DpdA) that replaces unmodified guanine bases with 7-deazaguanine in the DNA polymer (Fig. 1B). In some cases, such as in phage 9 g, the 7-deazaguanine is further modified to create the final modified base dG+ (13, 33).

Protein-mediated physical shielding may block action by HDR components of Type I and BREX systems. The bacteriophage P1 *d*efense *a*gainst *r*estriction (*dar*) system is composed of six proteins that are incorporated into the virion head–DarA, DarB, Ulx, DdrB, DdrA, and Hdf–that together confer protection from several Type I HDRM systems and BREX (423–425). Dar functions only *in cis* on DNA packaged in the virion (423): during coinfection experiments, Dar^–^ phages are restricted while coinfecting Dar^+^ phages are not (423), leading to the hypothesis that the Dar system may physically obstruct access to HDRM proteins to viral DNA during entry (426). However, recent data suggest that some Dar proteins are required for the incorporation of others into the P1 virion, complicating the interpretation of the phenotypes of dar mutants (424). The exact mechanism of Dar-mediated HDRM evasion remains unclear.

Finally, physical sequestration is possible. Jumbo phages in the family Chimalliviridae replicate their DNA within proteinaceous nucleus-like structures in the bacterial cytoplasm (427, 428). Certain nucleus-forming phages have been shown to be resistant to HDRM Types I and II, and a variety of CRISPR systems (427, 429). The nucleus-like structures produced by these phages are selectively permeable and exclude HDRM and CRISPR machinery from accessing the phage genome (427). Fusion of EcoRI or CRISPR nucleases to a phage nucleus resident protein allowed them to bypass the nuclear barrier and restore the antiphage activity of these immune enzymes, suggesting the nuclear shell is responsible for the broad resistance of these viruses to nuclease-based immunity (427). However, further work is required to fully understand the nature of restriction evasion by nucleus-forming phages, particularly prior to the formation of the nucleus structure early in the infection cycle (430).

#### The Trojan Horse defense: acquisition of protective methylation

Protective modification can be added by the MGEs themselves. Orphan modifiers (unaccompanied by HDR genes) may act to modify highly degenerate sites thus protecting against many distinct specificities. To prevent toxic effects on the host’s regulatory circuits these are expressed only upon exit from the cell. Many conjugal plasmids express facilitators of establishment upon entry in a region that is transcribed, and translated immediately before the double-stranded form appears (431, 432). One such facilitator family comprises homologs of a single-strand-specific adenine methyltransferase with very relaxed specificity (29). This system is protective against at least one Type II RE.

Temperate phages of *E. coli* and *Bacillus* also express modifying enzymes upon induction. The *Bacillus* temperate phages can protect against many RM systems with one enzyme by arraying multiple target recognition domains in a single polypeptide (31, 433, 434). Both the phage Mu DNA modification enzyme “Mom” (435) and the highly non-specific M.EcoGII (30) are silent in lysogens and toxic when artificially expressed. Many orphan MTases (mostly Type II) may regulate the viral replication cycles (34). For example, bacteriophage P1 requires Dam methylation (GmATC) at *pac* sites for proper packaging, making the P1 *dmt* methyltransferase essential for viral replication in Dam^–^ hosts (436). Bacteriophage T4 encodes a Dam methylase homolog which can methylate GATC and block the restriction of DNA *in vitro*, but for which the *in vivo* function remains unclear (437).

### Active antirestriction

Nucleotide-independent Type II HDRM systems have been found to be sensitive to passive anti-RM mechanisms (e.g., DNA hypermodification). However, direct inhibitors of such systems have not been found, possibly due to the variability of sequence recognition at the active sites of nucleotide-independent Type II REs precluding a general inhibitor able to bind diverse recognition domains. Nucleotide-dependent systems provide more points of leverage for interference. Limited testing suggests that the family relationships identified (described in “Transcriptional control configurations for Type IIP”) are reflected in the spectrum of anti-RM sensitivity. Promoting the acquisition of the correct methylation pattern may occur, as with Type IA RM responses to phage λ Ral (see “Transcriptional control configurations for Type IIP”).

#### Manipulation of cofactor pools

Depletion of cofactors required for HDRM activity may also contribute to restriction evasion by MGEs. S-adenosylmethionine is essential for Type I action in both HDR and HDM. Bacteriophage T3 (a T7-like virus) encodes an enzyme that degrades S-adenosylmethionine (SAM) via a lyase mechanism (not hydrolytically as once thought) (438). The gene is in the same genomic position as T7 Ocr (for overcome restriction) (439, 440; see further below). Early mutational studies suggested antirestriction function similar to Ocr for the T3 locus syntenic with T7 *ocr* (441). Despite the appealing hypothesis that degradation of SAM blocks Type I HDRM activity (being required for Type I HDR action), “SAMase” gene mutants can be isolated, which lack SAMase activity but maintain antirestriction function and vice versa (440, 442). Although an early study suggested that this could potentially be due to the physical interaction of the SAMase with a Type I HDRM complex resulting in inhibition of restriction (443), recent studies have clarified the two apparent functions of SAMase. Unexpectedly, T3 SAMase can inhibit the biosynthesis of SAM by binding to the host methionine S-adenosyltransferase (MAT) enzyme, which is required for SAM synthesis in *E. coli* (444, 445). Interestingly, BREX systems also require SAM for phage restriction, and the SAM degradation and MAT inhibition activities appear to act in concert to block BREX restriction (445). Further work is now required to understand whether these observations hold true for Type I HDRM systems as well.

#### Direct inhibition of REase activity

Phages and other MGEs also encode proteins that actively disable HDRM systems and other defense machinery in host cells. DNA mimics act by binding tightly where the enzyme-substrate would be. Bacteriophage T7 Ocr (289, 446), conjugative transposon Tn916 ArdA (32), and bacteriophage T4 Arn (447) all act as DNA mimics. Ocr is transcribed from the first open reading frame in the T7 genome during viral entry, whereas *ardA* is expressed from the early entry portion of conjugal plasmids (432).

The broadest demonstrated effect is of Ocr and relatives. These are typically carried by virulent phages and expressed before the full entry of viral DNA. They bind to and inhibit both restriction by the complete enzyme and modification by the methyltransferase. Ocr binds the active site of Type I HDRM complexes EcoK and EcoB to block activity (148, 289, 441). Both Type I families and BREX family systems (StyLT7II-BREX [279, 423] and EcoHS-BREX [272]) display strong responses. Notably, Type III enzymes are not responsive to Ocr. Ocr binds tightly to the Type I MTase core, blocking all action. Interestingly, transient expression from the native but UV-inactivated T7 phage relieved the restriction of a conjugal plasmid entry, leading to successful plasmid establishment and retransfer (448). Off-target action can also occur, enabling inhibition of vegetative RNAP in *E. coli*, another protein complex that surrounds the DNA. The authors suggest that the channel-forming transcription complex bound to DNA gives enough flexibility in the cavity to accommodate the rigid and bent Ocr dimer. Cell growth is shut down upon gratuitous induction of Ocr expression, concomitant with cessation of *lacZ* expression (449).

The ArdA family is distributed on many distinct conjugal plasmid families, all of which act *in vivo* to reduce restriction by Type I systems. ArdA of Tn916 acts by binding in the same region of the active site as Ocr (32, 450). Mutations in its dimerization interface selectively disable antimodification, sparing antirestriction (320).

The T4 Arn protein inhibits McrBC restriction of T4gt (451, 452). A structural model proposed binding to McrB as a DNA mimic (447). It may also act through effects on transcription mediated by a specific interaction with the DNA-condensing H-NS protein (447), which is known to affect transcription.

Phage λ also encodes a DNA mimic, Gam, that interferes with another nucleotide-dependent multi-subunit host defense, RecBCD. This inhibition prevents degradation of viral DNA upon entry before its sticky ends circularize the genome and also after cleavage by restriction endonucleases (453–455).

Ocr, ArdA, Arn, and Gam are all DNA mimic proteins specifically inhibiting anti-phage systems (32, 446, 447, 453) but are non-homologous, indicating multiple evolutionary origins of DNA mimic proteins in MGEs. One explanation for this phenomenon is that DNA mimics can exert pleiotropic pro-MGE effects. DNA mimicry by itself is not sufficient to create anti-restriction: McgB and QnrB target gyrase and do not inhibit Type I or BREX (272).

Direct inhibition without protein mimicry of DNA can be envisioned if the anti-RM action prevents assembly of the active form or causes mislocalization. This has not been shown, but candidates for such an activity include small proteins of phage and plasmids. It is not known whether the widespread Type I-directed ArdB family acts directly on the enzyme. *In vivo*, five family members strongly inhibited HDR but not HDM action, including representatives of four Type I families, a novel structural fold, that does not resemble a DNA mimic. No effect was found on R.EcoKI *in vitro*, nor on interference assembly from M2S + R2 (456).

Bacteriophage T4 encodes Stp, a short 26-mer peptide, which inhibits the Type IC restriction system EcoPrrI by an unknown mechanism, perhaps through physical interaction (457). Bacteriophage T4 also injects several proteins into cells along with its genome during entry, acting to both repress host immunity and modify host cell physiology (458). One of those proteins, IPI*, inhibits the action of some members of the Type IV GmrSD family (see “GmrSD family MDREs: DUF262+DUF1524” and “Anti-RM activities and host countermeasures”) that cleave DNA containing glycosylated cytosines (239). IPI* is basic but lacks overt structural homology to DNA (459). The locus encoding IPI* (*ip1*) is diverse among T4 relatives, and the inhibition of the target HDR enzymes is also variable.

#### REase degradation?

Some strategies for HDRM avoidance by MGEs can be envisioned which have not yet been experimentally identified. For example, degradation of immune sensors or effectors, especially by directing these proteins for degradation by cellular housekeeping machinery, is a common strategy for evasion of innate immunity employed by eukaryotic viruses (460). In bacteria, ClpXP is involved in the homeostatic regulation of HDRM systems via the degradation of Type I endonuclease subunits to prevent autoimmunity (187). Interestingly, some phages encode ClpXP homologs of unknown function, with the potential to act to inhibit the the HDRM system (426). Several other more putative mechanisms can be envisioned: Type I, Type III, and some Type IV systems function as complexes, and antirestriction factors interfering with the the assembly of the complexes could block the restriction function. RNA-based anti-CRISPR factors have been discovered encoded within phages and plasmids (461). Given that HDRM systems interact intimately with host and viral DNA, it is possible that similar nucleic-acid-based anti-restriction factors may be employed by MGEs to evade destruction by HDRM systems.

Given the enormous diversity of prokaryotic defense systems with predicted or known nucleic acid binding and/or manipulation activities (27, 324, 411, 450, 462), Ocr homologs and those of other MGE DNA mimics may have broader antidefense capabilities than are currently recognized. For example, Ocr, ArdA, and Arn can also interact with, and influence gene regulation by, the host H-NS protein (447, 463). H-NS and other small DNA-condensing proteins repress the expression of MGE genes including prophages (464).

### Host countermeasures against antirestriction

Restriction-modification systems are widespread among bacteria, representing the most common form of bacterial defense system currently appreciated, with ~80% of prokaryotic genomes encoding at least one system (8). The broad prevalence of HDRM across bacterial diversity has resulted in a range of strategies for evasion of restriction, as outlined above. Although factors for evasion of HDRM immunity can clearly be beneficial for MGEs, they also create new weaknesses that can be exploited by the host as signatures of MGE parasitism.

#### Layered defense

HDRM systems have been hypothesized to act as a first line of defense against MGE invasion. In a second layer of defense, several examples exist where secondary systems sense phage antirestriction proteins or their antirestriction activities and trigger cell death or growth arrest to prevent viral replication (411, 412). These “guardian” systems provide the host with layered immunity, utilizing kin-favoring suicide as a last resort in situations where the first line of defense has been disabled (27).

#### Detection of antirestriction proteins

The PARIS system, identified within a defense hotspot in the satellite phage P4, comprises two genes, *ariA* and *ariB* (411). These proteins assemble into a 425  kDa, supramolecular propeller-shaped complex, with six molecules of AriA and three subunits of AriB. The AriA function is to recognize the T7 Ocr antirestriction protein, which leads to the release of the AriB nuclease effector from the complex. Upon such activation, AriB alone forms a nuclease-active dimer that degrades host lysine transfer RNA (tRNALys), thereby blocking translation and leading to growth arrest or cell death (412). To avoid PARIS immunity, some T5 phages carry genes for their own tRNALys resistant to AriB cleavage (412). Escape mutants of phage T7 were selected which identified an F54V mutation in Ocr (411). PARIS reduces transformation by a plasmid encoding Ocr by over 100-fold but spares a plasmid encoding GFP. Notably, the OcrF54V can still confer evasion of the EcoKI Type I HDRM system (411), decoupling PARIS sensing from Ocr antirestriction activity.

Some HDRM systems are also coupled to accessory genes that have been hypothesized to function as toxins and drive abortive infection or growth arrest in response to phage activities (465). This observation clearly indicates that genomic synteny between HDRM systems and new defense systems revealed in recent studies (27, 324, 411, 450, 462) may provide important clues suggesting how different modes of prokaryotic immunity collaborate to restrict MGE propagation.

Beyond sensing antirestriction proteins, other countermeasures against HDRM evasion have been identified. Although phage DNA hypermodifications provide robust resistance against HDRM systems, they also provide the host with a unique molecular signature by which phage infection may be sensed (see “Possible translational control of Type II and assembly regulation of single subunit systems”). Type IV modification-dependent restriction enzymes such as GmrSD and McrBC directly sense and cleave phage DNA with modifications that allow phage to escape from other classes of HDRM. As phages create an incredibly broad array of modified DNA bases, it is easy to envision that an equally broad array of MDRE modification sensitivities may exist in nature.

#### Sensing REase inhibition

A well-studied example of this phenomenon is the *E. coli* accessory gene *prrC* (466, 467). PrrC is encoded by a gene embedded within a Type IC HDRM locus, *ecoprrI* (466). Upon inhibition of EcoPrrI by T4 Stp, the tRNALys anticodon nuclease activity of PrrC is activated to restrict host and viral protein synthesis (457). Interestingly, dTTP is important for the stability of active PrrC (468, 469), indicating that both EcoPrrI inhibition and an altered dTTP pool during phage infection (470) serve as triggers for PrrC activation. Interestingly, homologs of PrrC sense alternative triggers. *Acinetobacter baylii* RloC is activated by DNA double-strand breaks like those induced by T4 during host genome degradation, resulting in excision of the wobble base in tRNA and contained in the phage (471, 472).

#### Sensing of cofactor degradation

Several antirestriction countermeasures can be envisioned as pathogen-associated molecular patterns (PAMPs) for defense systems that have not yet been identified experimentally. Although MDREs detect and cleave hypermodified phage DNA for direct immunity, it is also possible that the accumulation of modified phage DNA could serve as a trigger for abortive infection systems. Likewise, conversion of the cellular CTP pool to 5hmdCTP prior to T-even phage DNA replication could provide another opportunity for phage detection through nucleotide pool monitoring. Atypical depletion of metabolites, such as HDRM cofactor degradation by T3 SAMase may also serve as signals of MGE invasion.

## CONCLUDING REMARKS AND OUTSTANDING QUESTIONS

Recent work using genome mining and functional selection approaches has revealed an incredible diversity of new defense systems targeting phages and other MGEs. Given the high prevalence of HDRM in bacterial genomes (8), and current evidence for collaboration with other systems like PARIS (411, 412), it is likely that we still have much to learn even about HDRM (the first known defense systems) from this newly appreciated network. For example, different classes of HDRM systems are positively and negatively associated with a variety of newly discovered defense systems (473). Investigation of such patterns is likely to reveal evolutionary relationships and potential synergy between HDRM and other forms of bacterial immunity yielding new mechanistic insights. Interactions are likely to affect both defense against MGE invasion and the shape of gene acquisition. Fresh eyes and a wealth of new knowledge and tools should enable insight into some aspects of HDRM biology presented here which remain shrouded in mystery.

Particular questions include how protective modifications are propagated during replication. For HDRM acting at asymmetric sites (see “Type IIS and Type III: asymmetric target sequences”), the *in vivo* solution to replication fork passage is mostly obscure; meanwhile, for PT systems [see “Phosphorothioate (PT) modification as protection and target”], incomplete modification is the rule, inviting further questions about maintenance in lineages. Current advances in methods to examine intracellular physiology (47, 48) should enable a better understanding of the physiology of interaction between entering DNA and the new cell.

Structural studies with modern cryo-EM and modeling approaches will very likely illuminate the interaction components of complex systems (see “Type IIS and Type III: asymmetric target sequences,” “Type I HDRM systems: asymmetric sites and two-strand modification,” and “Type IV systems: action when sites are modified”); these may be particularly fruitful for understanding how input signals are transformed into alternative outputs, with direct readout in the form of modification, cleavage, and translocation. Lessons learned might instruct understanding nucleases of recombination (RecBCD) and replicases as well as other defense functions.

Additional questions relate to modification-instructed mechanisms that abort MGE development without apparent DNA cleavage. Modification-protected BREX systems (see “BREX type 1: restriction without cleavage”) are the focus of the most recent attention and are likely to be illuminated in the near future. Potential targets of action may be found at a replication fork. Such answers might clarify the mechanism of the *in vivo* action of MDREs when *in vivo* cleavage is not apparent (see “Additional DNA binding domains in Type IV REs”).

More generally, MGE lifestyle categories may determine susceptibility to interference by the host: virulent or temperate phage, conjugal or hitchhiking plasmid, autonomous or integrating recombinase.
